# Cap1 forms a cyclic tetra-adenylate-induced membrane pore during the type III-A CRISPR-Cas immune response

**DOI:** 10.1101/2025.11.13.688252

**Published:** 2025-11-13

**Authors:** Puja Majumder, Clare W. Cahir, Cameron G. Roberts, Dinshaw J. Patel, Luciano A. Marraffini

**Affiliations:** 1 Structural Biology Program, Memorial Sloan-Kettering Cancer Center, New York, NY 10065, USA; 2 Laboratory of Bacteriology, The Rockefeller University, New York, NY 10065, USA; 3 Tri-Institutional PhD Program in Chemical Biology, Weill Cornell Medical College, Rockefeller University and Memorial Sloan Kettering Cancer Center, New York, NY 10065, USA; 4 Howard Hughes Medical Institute, The Rockefeller University, New York, NY 10065, USA

## Abstract

During type III CRISPR-Cas immunity in prokaryotes, RNA-guided recognition of viral (phage) transcripts stimulates the Cas10 complex to convert ATP into cyclic oligoadenylates. These act as signaling molecules that bind to CARF proteins and activate their effector domains. Here, we report the structure and function of the Cap1 effector, composed of a pair of transmembrane helices (TM1/2), a CARF-like (CARFL) domain and a domain of unknown function (DUF4579). Cryo-EM studies on apo- and ligand-bound states of Cap1 in glyco-diosgenin detergent revealed the formation of tetrameric complexes in both states, with one cyclic tetra-adenylate molecule bound in a pocket composed by the four CARFL domains. Binding of cA_4_ triggers conformational changes that widen an otherwise narrow pore formed by the four TM1/2 domains. *In vivo*, Cap1 activation results in membrane depolarization, a growth arrest of the bacterial host and the abrogation of the viral lytic cycle. Our findings reveal the mechanistic basis of membrane depolarizarion mediated by cyclic nucleotide signaling during the type III CRISPR-Cas response.

## INTRODUCTION

Prokaryotic clustered regularly interspaced short palindromic repeat (CRISPR) loci and their associated genes (*cas*) provide RNA-guided adaptive immunity against invading genetic elements such as bacteriophages^1^ and plasmids^2^. A unique feature of these loci is their ability to acquire short sequences from the invading genomes that are stored as “spacers” in between repeats of the CRISPR locus^1^. The CRISPR array of repeats and spacers is transcribed and processed into short CRISPR RNAs (crRNAs) that are loaded into Cas ribonucleoprotein complexes and used as guides for the recognition of complementary nucleic acids^3,4^. Depending on the *cas* gene content, CRISPR-Cas systems can be classified into six types^5^. Type III systems encode the crRNA-guided Cas10 complex^3,6^, which is activated upon recognition of a complementary RNA molecule produced by the invader. Target finding triggers two enzymatic activities in the Cas10 subunit of the complex: ssDNA degradation by the HD domain^7,8^ and cyclic oligoadenylate (cOA) synthesis using ATP molecules as substrate by the Palm domain^9,10^. The ssDNase activity is presumed to attack directly the phage’s genome and is sufficient to provide immunity when the target transcript is expressed early in the viral lytic cycle^11^. In contrast, when phage RNA is recognized late during infection, type III CRISPR-Cas immunity requires the activation of diverse accessory effector proteins containing CARF (CRISPR-associated Rossman fold) domains^12–14^. CARF domains form a pocket that accomodates cOA and are linked to an effector domain that is activated upon ligand binding. CARF effectors orchestrate a broader antiviral response by triggering diverse activities that are toxic both for host growth and viral replication, such as DNA and RNA degradation^15–19^, toxic nucleotide accumulation^20,21^, NAD^+^ depletion^22^ and membrane disruption or depolarization^23,24^. In this way, CARF effectors turn type III CRISPR-Cas immunity into an abortive infection mode of defense that sacrifices the infected host to prevent the spread of the phage into the rest of the bacterial population^11^.

While many CARF effectors have been identified, the full range of their antiphage defense mechanisms are still emerging. In addition, the structural basis for the molecular mechanisms underlying cOA activation of CARF effectors harboring transmembrane domains has remained unclear. Previous studies of two of these effectors, Cam1^23^ and Csx23^24^, revealed the formation of tetrameric structutures capable of binding cA_4_ and potent anti-phage activity (through membrane depolarization in the case of Cam1), yet the molecular basis and regulatory mechanisms underlying this immunity are not fully understood. Here we identified Cap1 (CRISPR-associated pore 1), a CARF effector from the *Parafilimonas terrae* type III-A CRISPR-*cas* locus composed of two transmembrane helices, a CARF-like domain and a domain of unknown function (DUF4579). We demonstrate that Cap1 is a cyclic tetra-adenylate (cA_4_)-activated membrane effector that provides antiphage defense during type III CRISPR immunity. We solved cryo-EM structures of apo- and cA_4_-bound Cap1 in glyco-diosgenin (GDN) detergent and monitored pore formation within the membrane spanning segments of tetrameric scaffolds. We found that upon cA_4_ binding to the CARF-like domain, the tetramer undergoes conformational changes that lead to pore opening and membrane depolarization, presumably due to the leakage of ions across the bacterial envelope. Depolarization halts the growth of the infected host, limiting phage replication and propagation. Our findings uncover structural details that establish a direct molecular link between cyclic nucleotide signaling and anti-phage defense through membrane depolarization by a CRISPR-associated membrane pore forming effector.

## RESULTS

### Cap1 induces growth arrest during the type III-A CRISPR-Cas response upon binding of cA_4_.

**Figure 1.**
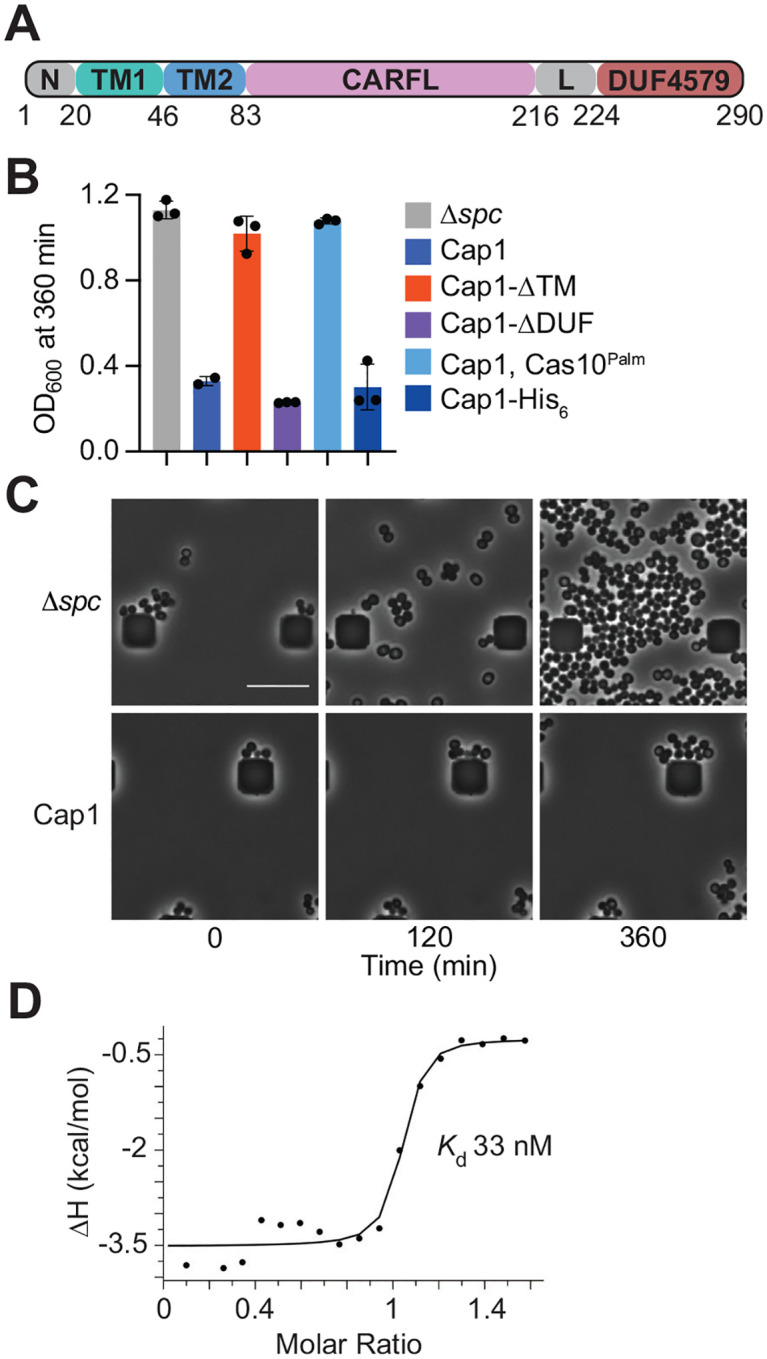
Cap1 mediates growth arrest upon activation of the type III CRISPR-Cas response. **(A)** The domain organization of Cap1 protein displaying the residue numbers for transmembrane segments (TM1, TM2), CARF-like domain (CARFL) and a domain of unknown function (DUF4579). **(B)** Growth of staphylococci carrying pTarget and different pCRISPR variants, measured as OD_600_ value at 360 minutes after the addition of aTc. Data show the mean of three biological triplicates +/− s.e.m. **(C)** Images from live microscopy of staphylococci carrying pTarget and different pCRISPR variants, taken at different times after the addition of aTc. **(D)** Binding of cA_4_ to the wild-type CARFL domain, measured by ITC; a *K*_d_ value of ~33 nM was estimated from the curve.

*P. terrae* habors a type III-A CRISPR-*cas* locus with a 26-spacer CRISPR array and encoding for the Cas1/2 integrase responsible for the insertion of new spacer sequences^25–27^, the Cas10-Csm crRNA-guided complex^3,7^, the Cas6 endoribonuclease that processes the CRISPR array transcript into crRNAs^4,28^, a CARF effector and two ring nucleases that share structural homology to characterized ring nucleases Csx3^29^ (PDB:6VJG) and DUF1874 (PDB:6SCF) (Fig. S1A). Close inspection of the CARF effector protein sequence indicated that it is composed of a short N-terminal domain (N) followed by two transmembrane segments (TM1 and TM2) and a CARF-like domain (CARFL, see below) connected to DUF4579 through a linker (L) segment (Fig. 1A). Due to its function (see below), we renamed this protein “CRISPR-associated pore 1”, Cap1. In the past, we studied the function of CARF effectors *in vivo* in staphylococci, in the context of the *Staphylococcus epidermidis* RP62 type III-A CRISPR-*cas* locus. This locus is highly similar to the native CRISPR system that encodes Cap1 in *P. terrae* (Fig. S1A). We replaced the endogenous CARF effector *csm6*^30^ of the staphylococcal locus with *cap1*, and cloned the resulting locus into the staphylococcal vector pC194^31^, generating pCRISPR. We also introduced truncated versions of *cap1* with deletions of TM1 and TM2 region (*cap1-ΔTM*) or of the DUF4579 domain (*cap1-ΔDUF*) (Fig. S1B). As controls, we generated versions of pCRISPR with mutations that inactivate both activities of Cas10: D586A-D587A in the Palm domain (*cas10*^*dPalm*^) to prevent the synthesis of cOA^9,10^ and H14A-D15A in the HD domain (*cas10*^HD^) to abrogate ssDNA degradation^8^ (Fig. S1B). In addition, a CRISPR locus without a targeting spacer (*Δspc*) was used as a non-targeting control (Fig. S1B). pCRISPR plasmids were transformed into *Staphylococcus aureus* RN4220 cells^32^ containing pTarget, a second plasmid producing an anhydrotetracycline (aTc)-inducible transcript complementary to the crRNA carried by the Cas10-Csm complex encoded by pCRISPR^30^. In this two-plasmid system, addition of the inducer triggers type III-A CRISPR-Cas immunity and the synthesis of cOAs in the absence of phage infection (degradation of pTarget by the ssDNase activity of Cas10 is avoided using the *cas10*^HD^ allele in pCRISPR^30^). To determine if Cap1 is toxic during the type III-A CRISPR response, as is the case for most other CARF effectors^15,20,22,23,30^, we followed the growth of cultures carrying both plasmids through measurement of optical density at 600 nm (OD_600_), 360 minutes after addition of aTc. Cap1 and Cap1-ΔDUF, but not Cap1-ΔTM, prevented culture growth (Figs. 1B and S1C), in a manner that depended on the presence of a crRNA guide to recognize the aTc-inducible target transcript and on the synthesis of cOA by the Palm domain of Cas10 (Figs. 1B and S1C). To investigate the consequences of Cap1 activation at the cellular level, we followed the induced cultures using live microscopy. While the non-targeting control cells grew undisturbed over time, cells in which type III-A CRISPR immunity was triggered proliferated at a very low rate (Fig. 1C).

Cas10 can synthesize different cOA, the most common being cyclic tetra- (cA_4_) and hexa-adenylates (cA_6_), but also cyclic tri-adenylates (cA_3_)^9,10^. To determine which one of these activates Cap1, we expressed a His-tagged version of the CARFL domain (residues 85–220; His_6_-Cap1-CARFL) in *Escherichia coli* cells and purified it using Ni^+2^ affinity chromatography followed by size exclusion chromatography (Figs. S1D–E). We used isothermal titration calorimetry (ITC) to determine the binding affinity of the purified protein for different cOA. We found that Cap1-CARFL-His_6_ binds cA4 with high affinity, with an estimated *K*_d_ value of 33 nM. No interaction with cA_6_ nor cA_3_ was detected (data not shown). Altogether, these results indicate that Cap1 is activated by the production of cA_4_ during the type III-A CRISPR-Cas response and leads to a growth arrest, but not death, of the host cell. Interestingly, Cap1 toxicity requires the transmembrane, but not the DUF4579, domain.

### Apo-Cap1 adopts a tetrameric topology

**Figure 2.**
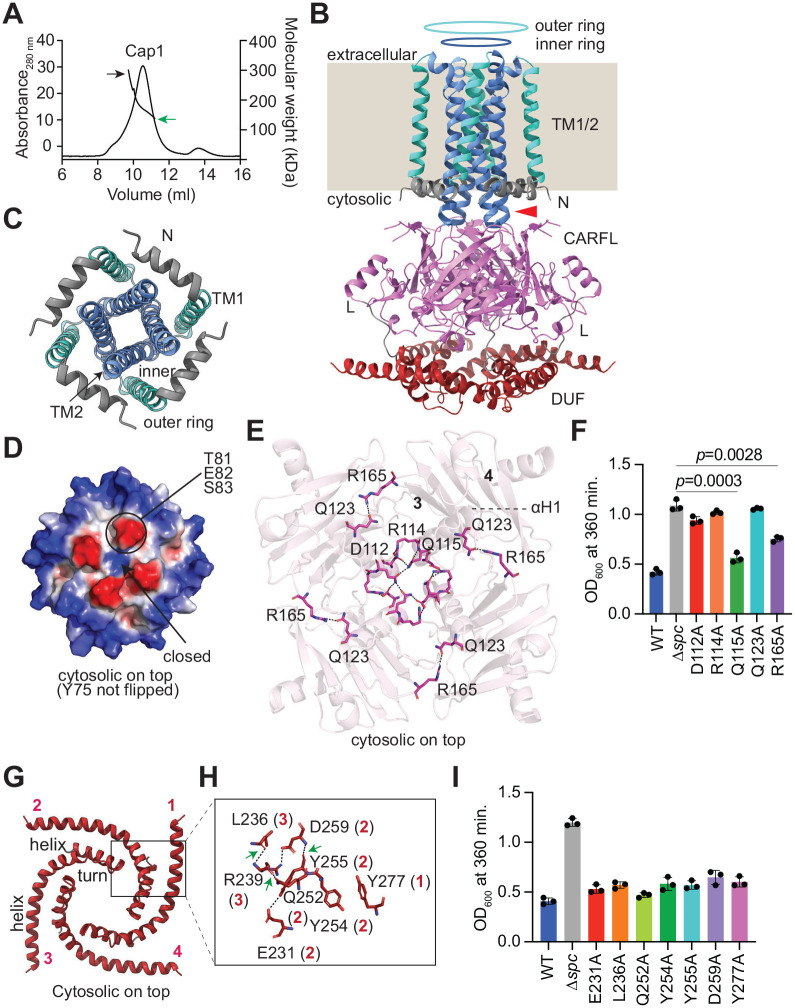
Apo-Cap1 tetramer structure. **(A)** SEC-MALS of apo-Cap1 protein. The major fraction elutes as a tetrameric form (green arrow); other fractions contain species with higher oligomeric states that are generated mostly due to mild aggregation of the protein (black arrow). **(B)** Ribbon diagram of the apo-Cap1 tetramer. TM1 and TM2 are displayed in green and blue and form outer and inner rings, respectively, that insert into the bacterial membrane (beige background, which replaces the density observed for the GDN detergent micelle). The cytosolic extension of TM2 is marked by a red arrow. The N-terminal segment (N, grey), CARFL domain (pink), the interconnecting linker (L, grey) and the DUF4579 domain (red) locate on the cytosolic side. **(C)** Cytosolic side view of the TM1 and TM2 domains of apo-Cap1 in green and blue, showing the formation of the outer and inner membrane rings, respectively. The N-terminal segment (N) is shown in grey. **(D)** Electrostatic surface representation of the cytosolic side view of the TM1 and TM2 domains, showing a closed pore. **(E)** Residues involved in CARFL tetramerization. D112, R114 and Q115 from αH1 of each monomer interact with each other at the center of the tetramer. R165 from the loop joining β strands 3 and 4 interacts with Q123 present in αH1. **(F)** Growth of staphylococci carrying pTarget and different pCRISPR variants harboring alanine substitutions of Cap1 residues shown in (E), measured as OD_600_ value at 360 minutes after the addition of aTc. Data show the mean of three biological triplicates +/− s.e.m. *p* values were obtained with two-sided t-tests with Welch’s correction. **(G)** Four-fold arrangement of DUF4579 helix-turn-helix in the apo-Cap1 tetramer (each monomeric helix-turn-helix is numbered 1 to 4). The black box indicates the interaction site of adjacent helix-turn-helices. **(H)** Residues involved in helix-turn-helix interactions with the region marked in (G) with a black box. **(I)** Growth of staphylococci carrying pTarget and different pCRISPR variants harboring alanine substitutions of Cap1 residues shown in (H), measured as OD_600_ value at 360 minutes after the addition of aTc. Data are mean of three biological triplicates +/− s.e.m. *p* values were obtained with two-sided t-tests with Welch’s correction.

To investigate the molecular mechanism behind Cap1’s toxicity, we expressed in *E. coli* a full version of Cap1 that harbors a C-terminal hexahystidyl tag (Cap1-His_6_). We confirmed that the addition of the tag did not alter the toxic properties of Cap1 (Figs. 1B and S1C). We then purified Cap1-His_6_ via Ni^+2^ affinity chromatography from the membrane fraction, in the presence of GDN detergent (Fig. S2A). First, we analyzed the oligomeric state of purified apo-Cap1 (in the absence of cOA ligands) using conjugate SEC-MALS, which rendered a somewhat broad peak indicative of tetramer formation (Fig. 2A, green arrow), along with the presence of some higher order species, mostly due to the mild aggregation propensity of the protein (Fig. 2A, black arrow). The peak fractions were collected to determine the atomic resolution structure of apo-Cap1 in GDN using cryo-EM. The collected images were separated in different representative 2D class averages (Fig. S2B). One class displayed a view from the cytosolic side that corroborated the tetrameric association suggested by SEC-MALS (Fig. S2B, image “*a*”). Another 2D class presented a side view of apo-Cap1, showing both the DUF and CARFL domain as well as some of the TM helices surrounded by the detergent micelle (Fig. S2B, image “*b*”; Fig. S2C). The high resolution cryo-EM map of apo-Cap1 established a tetrameric arrangement composed of cytosolic DUF and CARFL domains connected to the TM1/2 membrane-spanning domain (Figs. 2B, S2C–D). In this structure, TM1 crosses the bacterial membrane starting with its N-terminal end in the cytosolic side and then takes a sharp turn at the extracellular side to allow the re-entry of TM2 into the membrane (Fig. 2B). Notably, in the tetrameric arrangement of apo-Cap1, this sharp turn leads to the formation an inner ring of four TM2 helices surrounded by an outer ring of four TM1 helices (Figs. 2B–C). Interestingly, we observed extra density protruding out from one of the TM2 helices inside the inner ring (Fig. S2E, black box). Upon building the protein model we found that this extra density most likely corresponds to an alternate conformation of the Y75 side chain of one of the four TM2 helices (Figs. S2F–G). We then evaluated the electrostatic potential of the inner ring using Pymol and considered both conformations of the Y75 side chain. We found that in both cases the inner ring forms a closed pore with a positively charged cytosolic side that is surrounded by four negatively charged patches composed of residues T81, E82 and S83 in the TM2 segments (Figs. 2D, S2H). These residues are part of a short cytosolic extension of TM2 that separates the membrane from the cytosolic CARFL domains (Fig. 2B, red arrow).

The CARF domain of apo-Cap1 (Cap1-CARF) assembles in a tetrameric conformation, with four αH1 helices at the center of the tetramer (Figs. 2E, S2I) that form a positively charged pocket with 4-fold symmetry (Fig. S2J). In most CARF effectors such pocket is formed by a dimer of CARF domains and accommodates the cOA second messenger^12–14^. In contrast, our cryo-EM data suggests that this effector captures its ligand using a tetrameric binding pocket, a highly unconventional arrangement. A more detailed structural comparison of Cap1-CARF with the canonical CARF domain of Cad1, a previously studied CARF effector^20^, further differentiated both domains. A β sheet that is sandwiched between two pairs of α helices in conventional CARF domains (αH1-αH2 and αH3-αH4, Fig. S2K) remains between two α helices (αH1 and αH2, Fig. S2L) in Cap1-CARF, making this domain slightly smaller in size. In addition, a β-sheet formed by five parallel and one antiparallel β-strands in Cad1’s CARF (Fig. S2K) is formed by two parallel (β1, β3) and three antiparallel (β2, β4, β5) strands in CARFL (Fig. S2L). Due to these substantial differences, we decided to name the Cap1-CARF domain “CARF-like” (CARFL). The CARFL tetramer is stabilized by residues D112, R114 and Q115 located in αH1 that interact with each other and with equivalent residues in the αH1 of an adjacent monomer, as well as by the interaction between Q123 in αH1 and R165, situated in a loop joining β-strand 3 and 4 from a contiguous CARFL region (Fig. 2E). To assess the importance of tetramer formation and/or stability for Cap1 function, we generated single alanine substitutions of these residues and tested for the ability of Cap1 to cause toxicity upon activation of the type III-A CRISPR response. We found that while Q115A and R165A mutations did not alter Cap1-mediated toxicity, substitution of D112, R114 and Q123 for alanine abrogated the growth arrest (Figs. 2F, S2M). Given that Q123 interacts not only with R165 but also with the backbone of F132 (Fig. S2N), it is possible that the Q123–F132 interaction that remains in the R165A mutant is sufficient to stabilize the CARFL tetramer. Likewise, it is conceivable that in the Q115A mutant the interactions between D112 and R114 (Fig. S2N) are able to maintain the tetrameric structure.

The cryo-EM structure of the DUF4579 domain of the apo-Cap1 revealed that it adopts a helix-turn-helix motif arranged in a tetraskelion-like shape in the tetramer (Fig. 2G). This shape is stabilized by interactions both within and between adjacent helix-turn-helices: E231, Q252, Y254, Y255 and D259 from monomer 2 interact with each other, with Y277 from monomer 1 and with L236 and R239 from monomer 3 (Fig. 2H). In contrast, we did not detect contacts between DUF4579 and CARFL resisues. We tested the importance of the DUF4579 residues presumably involved in the interactions described above throught the introduction of alanine substitutions. Interestingly, none of the mutations limited the Cap1’s ability to induce growth arrest during the type III-A CRISPR-Cas response (Figs. 2I, S2O). This is in line with our previous result that showed a similar lack of effect on Cap1’s function for the deletion of the entire domain (Cap1-ΔDUF, Fig. 1B).

### The cA_4_-Cap1 tetramer binds a single cA_4_ molecule.

**Figure 3.**
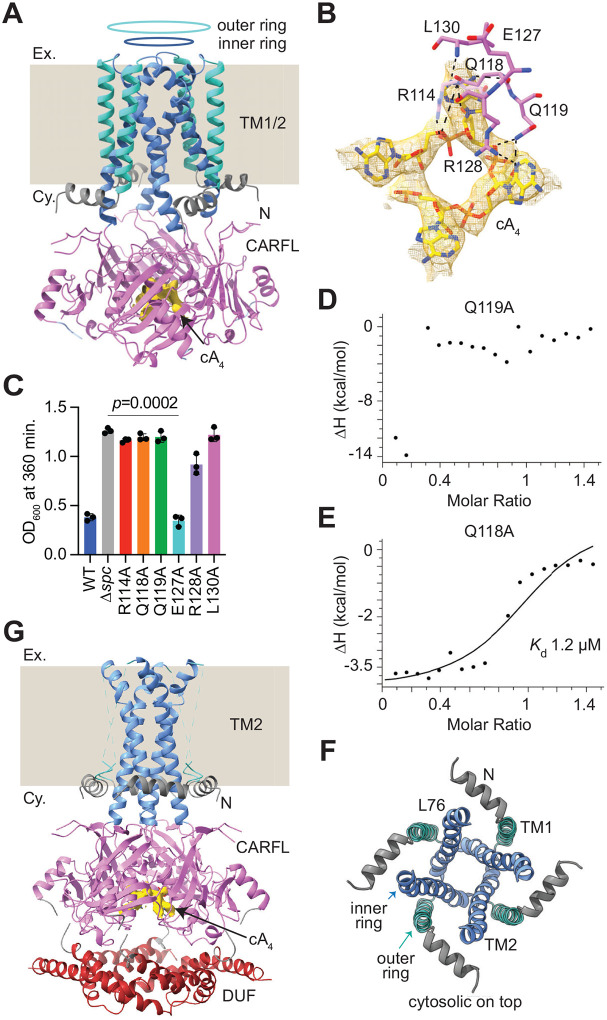
cA_4_-Cap1 structure. **(A)** Ribbon diagram of the cA_4_-Cap1 tetramer. TM1 and TM2 are displayed in green and blue and form outer and inner rings, respectively, that insert into the bacterial membrane (beige background, which replaces the density observed for the GDN detergent; “Ex.”, extracellular space, “Cy.” cytosol). The N-terminal segment (N, grey) and the CARFL domain (pink) locate on the cytosolic side. The density for the DUF4579 domain was not visible. **(B)** Residues of one CARFL monomer (pink sticks) that interact with cA_4_. The density for the cA_4_ molecule is shown in yellow mesh representation, at contour level ~9 RMS from the C1 symmetry cA_4_-Cap1 map. **(C)** Growth of staphylococci carrying pTarget and different pCRISPR variants harboring alanine substitutions of Cap1 residues shown in (B), measured as OD_600_ value at 360 minutes after the addition of aTc. Data show the mean of three biological triplicates +/− s.e.m. *p* values were obtained with two-sided t-tests with Welch’s correction. **(D)** Binding of cA_4_ to the CARFL^Q119A^ domain, measured by ITC. **(E)** Binding of cA_4_ to the CARFL^Q118A^ domain, measured by ITC; a *K*_d_ value of ~1.2 μM was estimated from the curve. **(F)** Cytosolic side view of the TM1 and TM2 domains of cA_4_-Cap1 in green and blue, showing the formation of the outer and inner membrane rings, respectively. A kink is observed in each TM2 helix at residue L76. The N-terminal segment (N) is shown in grey. **(G)** Ribbon diagram of the cA_4_-Cap1 tetramer with a visible DUF4579 domain. TM2 helices are displayed in blue and form the inner ring that inserts into the bacterial membrane (beige background, which replaces the density observed for the GDN detergent; “Ex.”, extracellular space, “Cy.” cytosol). The N-terminal segment (N, grey) and the CARFL domain (pink) locate on the cytosolic side. The density for the outer ring of TM1 helices could not be modelled.

To gain mechanistic insights into Cap1’s activation, we solved cryo-EM structures in the presence of cA_4_. The obtained images were separated into two distinct classes. The cryo-EM map of the first class at high (2.9 Å) resolution showed formation of a tetrameric complex, but without a visible DUF4579 domain (Figs. 3A, S3A). To ensure that this domain was not cleaved after cA_4_ addition, we compared the size exclusion chromatograms of apo- and cA_4_-bound Cap1. Since we obtained a very similar elution profile for both proteins (Fig. S3B), we concluded that the DUF4579 domain is still present after cA_4_ binding, but cannot be detected most likely due to high flexibility and/or structural heterogeneity.

We observed one cA_4_ bound per tetramer, with the density corresponding to cA_4_ buried inside the CARFL tetrameric pocket, at the center of 4-fold symmetry, surrounded by αH1 helices (Figs. 3A, S3C–D). This is unusual for most cA_4_-CARF interactions, where cA_4_ binds to the pocket at the interface of a CARF dimer, being positioned on top of the two αH4 helices, as shown in the previously studied cA_4_-Cad1 structure (Fig. S3E)^20^. In addition, the adenine bases of the ligand are pointing upwards towards the cytosolic direction, rather than being positioned in the same plane as rest of the molecule (Figure S3F). Although this unconventional binding of the cA_4_ molecule was not observed in any of the previously solved structures of CARF/SAVED/Csx3 domains, a similar arrangement was observed for the cA_4_ molecule bound to tetrameric cytosolic domain of Csx23^24^ (Figs. S3G–H). We refined the cryo-EM map with C1 symmetry followed by C4 symmetry to improve the resolution. However, in both cases the CARFL domains were well resolved, and the map correspond to the cA_4_ molecule was similar (Figs. S3C–D); therefore, we used the C1 symmetry cA_4_-Cap1 map for further analysis of the ligand. In this structure, cA_4_ interacts with R114, Q118, Q119, E127, R128 and L130 residues (Fig. 3B). With the exception of E127A, alanine substitutions of these residues impaired the ability of Cap1 to mediate a growth arrest (Fig. 3C). We also purified Cap1-CARFL-His_6_ domains carrying the Q118A and Q119A mutations (Figs. S1D, S3I–J) and performed ITC experiments to measure cA_4_ binding directly. We observed severe reductions in ligand binding, with the Q119A substitution completely abrogating cA_4_ interaction (Fig. 3D) and the Q118A mutation decreasing binding by more than two orders of magnitude (*K*_d_ ~ 1.2 μM; Fig. 3E). Altogether these results corroborate the importance of CARFL residues for ligand binding and Cap1 activation.

The TM domains of the cA_4_-Cap1 complex maintained the outer TM1 and inner TM2 ring structure. However, in the inner ring the four TM2 helices were slightly bent at the L76 residue, making the cytosolic entry of the pore (Fig. 3F) wider than that observed for the apo state (Fig. 2D). Below, we discuss in more detail the conformational changes of the pore.

The cryo-EM map of the low resolution class (3.6 Å) showed the DUF4579, the CARFL and TM2 domains, however the TM1 helices were not resolved (Figs. 3G, S3K). The cA_4_ binding site and the arrangement of cA_4_ in this structure were similar to those observed in the high resolution cA_4_-Cap1 complex, with comparable interacting residues (Fig. S3L). In addition, the DUF4579 domain also adopted a similar tetrameric conformation as in apo-Cap1 (Fig. S3M).

### cA_4_-mediated opening of Cap1’s transmembrane pore causes membrane depolarization

**Figure 4.**
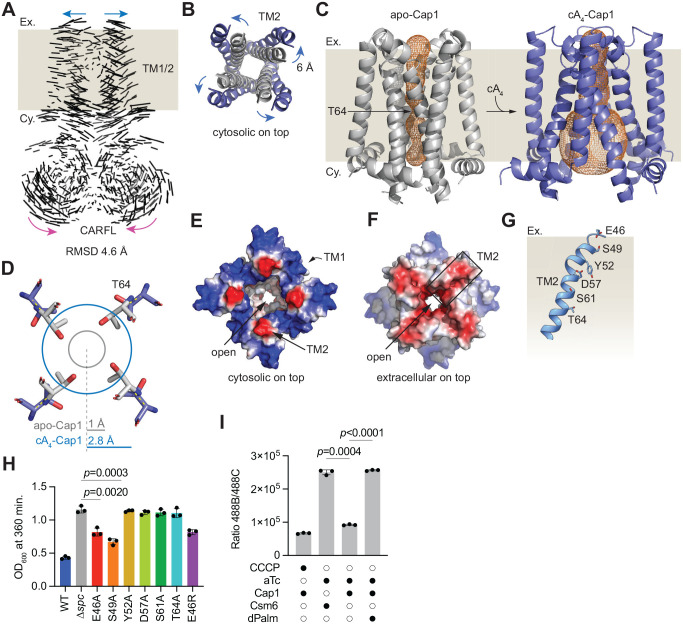
Pore opening upon cA_4_ binding causes membrane depolarization. **(A)** Superposition of apo- and cA_4-_-bound Cap1 (without visible DUF4579) structures shows that ligand binding promotes an outward movement of the TM1/2 helices (blue arrow) and an inward movement of the CARFL domain (pink arrow). The bacterial membrane is represented by the beige background; “Ex.”, extracellular space, “Cy.” Cytosol. The overall Cα RMSD between structures is 4.6 Å. **(B)** Cytosolic view of the outward movement (blue arrows) of TM1 inner ring helices. Helices in apo-Cap1 and cA_4_-Cap1 are shown in grey and blue, respectively. **(C)** Analysis of the pore formed by the TM1/2 domain using Mole2.5 software, represented as the orange mesh inside the TM2 ring. Apo-Cap1 structure shown has the Y75 side chain not flipped. The bacterial membrane is represented by the beige background; “Ex.”, extracellular space, “Cy.” Cytosol. The narrowest part of the pore is formed almost at the middle of TM2 and is surrounded by four T64 residues. The pore opens towards the cytosolic side of TM2 in the cA_4_-bound conformation of Cap1 (without visible DUF4579). **(D)** Cross-sectional view of the T64 side chains within the pore in the apo- and cA_4_-bound forms of Cap1 in grey and blue sticks, respectively. The closed (grey) and opened (blue) pore circunferemces and radii at the cross-section, calculated with Mole2.5 software, are indicated. **(E)** Electrostatic surface representation of the cytosolic side view of the TM1 and TM2 domains, showing an opened pore. The positively charged patches are formed by R10 and R11 residues from the N-terminal segment and K21 and R22 residues from the TM1 helices. The electronegative patch is formed by T81, E82 and S83 residues present at the cytosolic extension of TM2 helices. **(F)** Electrostatic surface representation of the extracelluar side view of the TM1/2 domain, showing an opened pore with an electronegative lining. **(G)** Negatively charged and polar residues located in the inner TM2 helix, displayed in stick representation. **(H)** Growth of staphylococci carrying pTarget and different pCRISPR variants harboring alanine substitutions of Cap1 residues shown in (G) (and also E46R), measured as OD_600_ value at 360 minutes after the addition of aTc. Data are mean of three biological triplicates +/− s.e.m. *p* values were obtained with two-sided t-tests with Welch’s correction. **(I)** Quantification of green/red fluorescence ratio (488B/488C channels) obtained using flow cytometry of staphylococci carrying pTarget and different pCRISPR variants stained with DiOC_2_ (3) after induction of cA_4_ synthesis via aTc treatment of the cultures. Data are mean of three biological triplicates +/− s.e.m. *p* values were obtained with two-sided t-tests with Welch’s correction.

Structural alignment of the apo- and cA_4_-bound Cap1 tetramers revealed ligand-induced conformational changes in the TM1/2 and CARFL domains (Fig. 4A), as well the loss of resolution of the DUF4579 domain. The most significant of these changes is a 6 Å outward shift of the inner TM2 helices (as viewed from the cytosolic side, Fig. 4A–B, blue arrows) that leads to the opening of the pore formed by the TM1/2 domains. We used the Mole2.5 software^33^ to quantify the pore opening and found a constricted region around the middle of the membrane spanning segment, surrounded by T64 residues located in TM2, that widens towards the cytosolic side in the presence of bound ligand (Fig. 4C). A similar pore constriction was determined for the apo-Cap1 structure in which Y75 side chain is flipped-in, althouth the length of the pore cavity is approximately 1 Å shorter (Fig. S4A). Structural measurements revealed that, upon cA_4_ binding, an outward movement of T64 residues extends the pore radius from 1.0 Å to 2.8 Å (Fig. 4D). We also analyzed the electrostatic nature of the pore in the cA_4_-Cap1 complex. On the cytosolic side, the pore is surrounded by four negatively charged patches formed by the cytosolic extension of TM2 (T81, E82 and S83), and the outer ring is positively charged due to the presence of residues from TM1 (K21 and R22) and N-terminal domain (R10 and R11) at the cytosolic side of the pore (Fig. 4E). In contrast, on the extracellular side the pore is negatively charged, both on the region surrounding the pore opening as well as inside the pore (Fig. 4F). This is a consequence of the existence of negatively charged and polar residues of TM2 lining the pore: E46, S49, Y52, D57, S61 and T64 (Fig. 4G). We generated alanine substitutions of these residues and found that Y52A, D57A, S61A and T64A completely impaired Cap1’s-mediated toxicity in staphylococci, with E46A and S49A displaying a less drastic effect (Figs. 4H, S4B). We also introduced a positive charge in position 46 (E46R mutation), however this substitution did not have a more profound effect on cell growth (Figs. 4H, S4C). These results emphasize the importance of the pore lining residues located in the TM2 domain for Cap1 activity.

Given that cA_4_ binding to Cap1 induces a conformational change that opens a pore formed by the transmembrane helices TM1/2, we hypothesized that if these domains are indeed embedded in the bacterial host membrane, Cap1’s activation during the type III-A CRISPR-Cas response could lead to the passage of ions into or out of the cell. Such transfer could either disrupt the membrane potential and/or integrity, leading to membrane depolarization, as it is the case for the previously characterized CRISPR associated effectors Csx28^34^ and Cam1^23^. To test this, we induced synthesis of the cA_4_ ligand using aTc, as in the toxicity assays experiments, and stained staphylococci with the membrane potential indicator dye 3,3’-dietyloxacarbocyanine iodide (DiOC_2_(3)). The DiOC_2_(3) dye emits green and red fluorescence, with a shift towards red emission in the presence of higher membrane potentials^35^. Therefore, membrane potential can be measured independent of cell size by calculating the red/green fluorescence intensity ratios^35^. We used carbonyl cyanide *m*-chlorophenylhydrazone (CCCP), a common membrane potential disruptor, as a positive control^35^. As a negative control we used cells expressing Csm6, a CARF effector that does not affect the host membrane^30^, instead of Cap1. Flow cytometry analysis of the cells treated with DiOC_2_(3) showed that Cap1, but not Csm6, activation decreased the red/green fluorescence ratio only in the presence of a functional Palm domain capable of synthesizing cA_4_ (Figs. 4I and S4D). Collectively, these results support a model in which cA_4_ signals generated by the Cas10 complex upon target recognition, when bound by the CARFL domain of the transmembrane Cap1 tetramer, induce a conformational change that results in the opening of a pore across the bacterial membrane. This in turn causes membrane depolarization and cell toxicity, preventing the normal growth of the host and the replication of the infecting virus.

Regarding conformational changes detected for the other domains of Cap1 upon ligand binding, we found that the αH1 helices of the CARFL domain showed an inward shift of 3.5 Å (Figs. 4A and S4E, magenta arrows). This is an opposite movement to that experienced by the TM1/2 domains and leads to the formation of a compact cA_4_ binding pocket. A major difference between apo and cA4-bound Cap1 structures was the absence of a defined DUF4579 domain in the latter. We used Alphafold3 (AF3) to corroborate this observation. The best four AF3 models of apo-Cap1 yielded similar structures to the one obtained by cryo-EM, with a good alignment of the four predicted DUF4579 domain structures (Fig. S4F). Superposition of the cryo-EM and top AF3 DUF4579 domain structures (red and silver, Fig. S4G) showed a good agreement between the two, with a Cα RMSD of 0.5 Å. In contrast, the best four AF3 models for the cA_4_-Cap1 tetramer displayed a good alignment through the TM1/2 and CARFL domains, however the the DUF7549 domain remained completely miss-aligned and highly heterogeneous (Fig. S4H). Therefore, AF3 modeling supports our hypothesis based on cryo-EM results that cA4 binding generates a conformational change that leads to a disordered DUF4579 domain.

Finally, superposition of the cA_4_-bound Cap1 structure with a visible DUF4579 domain to the apo form revealed minimal conformational changes in the CARFL and TM2 domains, with a Cα RMSD of 1.3 Å (Fig. S4I). This is a better structural alignment for these domains than that between both cA_4_-Cap1 structures (with and without visible DUF4579 domain), which has a larger Cα RMSD of 2.4 Å (Fig. S4J). This suggests that in the presence of the DUF, the CARFL region bound to cA_4_ is not fully compacted and the TM2 domain retains a conformation more similar to that of the apo form (Figs S4J–K), with a constricted pore (Fig. S4L). In conclusion, we observed that the cA_4_-bound Cap1 structure with a visible DUF4579 exhibits minimal conformational changes compared to the apo form.

### Cap1 mediates immunity against phage infection

**Figure 5.**
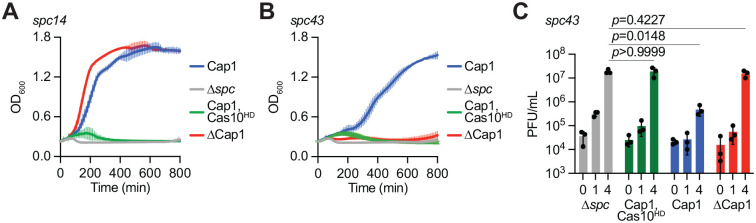
Cap1 mediates anti-phage immunity. **(A)** Growth of staphylococci carrying different pCRISPR constructs targeting the *gp14* transcript produced by the ϕNM1γ6 phage, measured as OD_600_ after infection at MOI ~5. Data are mean of three biological triplicates +/− s.e.m. **(B)** Same as in (A), but testing the immunity of pCRISPR constructs programmed to target the *gp43* transcript. **(C)** Number of plaque-forming units (PFU) in staphylococcal cultures harboring different pCRISPR constructs targeting the *gp43* transcript, at the indicated times after infection with ϕNM1γ6 at MOI ~1. Data are mean of three biological triplicates +/− s.e.m. *p* values were obtained with two-sided t-tests with Welch’s correction.

Next we tested the role of Cap1 during the type III-A CRISPR-Cas response against phage infection. As a consequence of RNA targeting by the Cas10 complex^3,8^, this response depends on when the target transcript is produced during the viral lytic cycle^11,16,36^. CARF effectors are typically dispensable for defense when the target is recognized early after infection, but absolutely essential when the phage transcript is recognized by the Cas10 complex in the latest stages of infection^11,15,20,22,23^. We therefore programmed the CRISPR array of the pCRISPR plasmid with two spacers (*spc14* and *spc43*) producing crRNAs complementary to early- and late-expressed transcripts (*gp14* and *gp43*, respectively) from staphylococcal phage ϕNM1γ6. Each spacer was introduced into two different genetic backgrounds for the *cas10* gene: wild-type and cas10^HD^, carrying mutations in the nuclease active site (H14A, D15A) that prevent ssDNA cleavage^8^ (Fig. S1B). This mutantion forces the type III-A immune response to rely exclusively on the CARF effector^11,30^, and therefore allows us to measure the ability of Cap1 to defend the host from infection. As a negative control we generated pCRISPR plasmids harboring *spc14* or *spc43* but without encoding a CARF effector (*Δcap1*, Fig. S1B). We infected staphylococci harobring the different pCRISPR plasmids with ϕNM1γ6 at a multiplicity of infection (MOI) of ~5 and determined culture survival by measuring OD_600_. When programmed to target the early-expressed transcript (*gp14*), the type III-A CRISPR-Cas response required ssDNA degradation by the Cas10 complex, but not Cap1 (Fig. 5A). In contrast, when the target RNA is recognized late in the viral lytic cycle (*gp43*), Cap1 was necessary to support immunity and also required the nuclease activity of Cas10. Neither of these activities alone was sufficient to enable culture growth after infection (Fig. 5B). We corroborated these results by evaluating the effect of type III-CRISPR-Cas immunity on phage propagation, enumerating plaque-forming units (PFU) on lawns of staphylococci carrying different pCRISPR(*spc43*) plasmids. We found a significant reduction in PFU only when hosts carried active Cap1 and Cas10, but not with either of these immune effectors alone (Fig. 1C). These results demonstrate that, as previously found for other CARF effectors^20,22,23^, Cap1 is required for a successful type III-A immune response that is activated late in the viral lytic cycle.

## DISCUSSION

In this study we elucidated the structure and mechanism of action of Cap1, a type III CARF effector. We show that Cap1 adopts a tetrameric architecture, with eight hydrophobic helices forming a pore that most likely spans the bacterial membrane and four CARF domains that form a pocket for a single cA_4_ molecule. In the absence of ligand the pore is closed, but opens upon binding of the second messenger produced after the recognition of phage infection that triggers the type III-A CRISPR-Cas response. Cap1 activation leads to membrane depolarization, presumably due to the passage of ions accross the open pore. Membrane depolarization is toxic to the host, which stops growing to prevent phage propagation.

Previous work has uncovered three transmembrane effectors that participate in the CRISPR-Cas immune response. VanderWal *et al*. reported that Csx28, which is associated with type VI-B CRISPR-Cas systems^34^, displays an octameric configuration that forms a pore across the bacterial membrane. Csx28 is required for anti-phage defense, presumably through induction on membrane perturbations, but since type VI systems do not synthesize second messengers, it is not clear how it is activated and whether there is a mechanism to open and close the pore. Baca *et al*. described Cam1, a CARF effector linked to a short hydrophobic helix that localizes to the membrane^23^. Cam1 is part of a type III-A CRISPR-Cas system and is activated by cA_4_ upon crRNA-guided recognition of the infecting phage transcripts to cause membrane depolarization and prevent the viral lytic cycle, a similar activity to that of Cap1. Although AF3 models predict the formation of a tetrameric pore across the membrane, the structure of full-length Cam1 in either apo- or cA_4_-bound states is not available, and it is not known how cA_4_ binding leads to its activation. Finally, Grüschow *et al*. charachterized Csx23, an effector associated with a type III-B CRISPR-Cas system with a unique cA_4_ binding domain fused to a short transmembrane domain^24^. Using Pulse Dipolar Electron Paramagnetic Resonance Spectroscopy (PDS) to measure conformational changes, it was shown that cA_4_ binding to the purified full-length protein caused changes in the PDS signal that are consistent with structural heterogeneity in the transmembrane region. However, in the absence of structural data, the precise nature of the changes triggered by ligand binding, as well as how these changes lead to Csx23 toxicity, is not known. Outside of the CRISPR-Cas response, a group of effectors from cyclic oligonucleotide-based antiphage signalling systems (CBASS) contain transmembrane helices (Cap14, Cap15 and Cap16) that bind cyclic nucleotide second messengers to oligomerize and disrupt the membrane of infected cells^37^. As in the other examples cited above, the conformational changes of the transmembrane domains that occur upon ligand binding are not known. In contrast to these previous studies, our characterization of Cap1 shows with atomic resolution how cA_4_ binding to the CARFL domain results in an outward movement of the transmembrane helices that opens a pore, causing membrane depolarization to compromise the infected host and thus prevent the propagation of the invading virus.

Cryo-EM analysis uncovered that Cap1’s CARF domain, which we called CARF-like or CARFL, diverges from conventional CARF domains both in protein fold and oligomeric assembly. While most of these structures typically form dimers with two-fold symmetry and bind one cA_4_ molecule at the dimer interface, CARFL assembles into a tetramer with four-fold symmetry and binds a single cA_4_ molecule at the central tetrameric interface – revealing a fundamentally different mode of ligand recognition. Notably, the central pore traverses not only the TM segments, but also the CARFL domains including the central hole of the bound cA_4_ second messenger. The previously characterized C-terminal cA_4_-binding domain (CTD) of Csx23 and the CARFL domain of Cap1 adopt a similar tetrameric arrangement that binds a single cA_4_ molecule (Fig. S3C,D,G,H)^24^. Although both share a similar oligomeric arrangement, the Csx23 CTD comprises two antiparallel β-strands linked to a third by two α-helices, whereas the Cap1 CARFL domain forms a mixed β-sheet of two parallel and three antiparallel strands flanked by two α-helices, yielding a structurally distinct and bulkier domain architecture. (Fig. S2L and S3H). The uncovering of these two structures that significantly diverge from canonical CARF domains suggests the possibility that many more yet uncharachterized cOA-binding domains exist.

Cap1 also contains a domain of unknown function, DUF4579, that adopts a tetrameric fold, with each protomer forming a helix-turn-helix motif. This domain is connected through a linker to CARFL and caps it from the bottom. There are no detectable side chain interactions between CARFL and DUF4579 domains in the apo-Cap1 cryo-EM structure. Intriguingly, DUF4579 appears to be disordered in the cA_4_-bound state of Cap1, suggesting increased flexibility. Consistently, AF3 predictions show high structural heterogeneity for this domain when cA_4_ is included during modeling, reinforcing the idea that cA_4_ binding induces conformational variability or destabilization of this region. Neither mutations that disrupt the tetrameric fold of this domain nor its complete deletion affected Cap1’s toxicity. These observations suggest that the DUF4579 domain may either be dispensable for Cap1’s function or play a role in stabilizing the closed pore conformation of the apo state. Interstingly, we also identified a cA_4_-bound Cap1 class comprising a small number of particles in which the DUF4579 domain is visible, and the membrane pore remains constricted, i.e. not much different than the apo-Cap1 structure. The function of the DUF4579 domain represents an open area of investigation; it is conceivable that it can play a specific role in its native host or when bacteria are growing in different media. Future studies hopefully will elucidate the importance of DUF4579 and other unknown domains linked to type III CRISPR-CARF effectors.

## METHODS

### Bacterial growth

*Staphylococcus aureus* strain RN4420^32^ was grown in brain heart infusion (BHI) medium at 37 °C, supplemented with chloramphenicol at 10 μg ml^−1^ for maintaining pCRISPR and erythromycin at 10 μg ml^−1^ for maintaining pTarget. 5 μM CaCl_2_ was supplemented in phage experiments unless indicated otherwise.

### Plasmid construction

The plasmids, oligonucleotides and cloning strategies for generating the plasmids used in this study are detailed in Supplementary File S1. Cap1 coding sequence, NCBI GenBank reference sequence WP_090655733.1 from *Parafilmonas terrae* bacterium, was synthesized by Genewiz.

### Growth curves

For *in vivo* Cap1 toxicity induction, biological replicates of RN4220 overnight cultures containing pTarget and pCRISPR are diluted 1:100, outgrown for about 75 min and normazlied for optical density. Cells are then seeded in a 96-well plate. To induce targeting, 125 ng ml^−1^ of aTc is added to the appropriate wells. Absorbance at 600 nm is then measured every 10 min by a microplate reader (TECAN Infinite m200 PRO).

For *in vivo* antiphage immunity, cells containing various pCRISPRs were launched in triplicate overnight, diluted 1:100, outgrown for about 75 min and normalized for optical density. Cells were seeded into a 96-well plate. ΦNM1γ6 was added at the specified MOIs, and optical density measurements were taken every 10 min.

### Quantification of phage plaques

To obtain plaque-forming unit (PFU) counts over time from cultures infected with phage, *S. aureus* cultures containing various pCRISPRs were launched overnight, diluted 1:100 and outgrown for about one hour. Cells in media supplemented with 5 mM CaCl_2_ were then infected with phage ΦNM1γ6 at an MOI of approximately 5, and an aliquot, filtered through a 0.45 μm membrane (Pall Acrodisc), was taken shortly after to obtain plaque-forming units at time 0. The cultures were then incubated further, with aliquots taken at one and four hours. For phage plaquing assays, indicated phage stocks were plaqued on lawns of *S. aureus* containing the indicated constructs, or cells lacking an introduced pCRISPR plasmid to measure PFU changes over time, with 10-fold serial dilutions for every spot in a lane.

### Flow cytometry

For our membrane depolarization studies, colonies of *S. aureus* containing pTarget and the specified pCRISPR were launched in liquid culture overnight. The next day, cells were diluted 1:100 and grown out for about 75 min and normalized to 10^7^ cells ml^−1^ in PBS. These cultures were then split into different subcultures and treated with either 125 ng ml^−1^ aTc or 1.7 μM CCCP (Thermo Fisher). These subcultures were incubated in shaking conditions at 37 °C for 30 min followed by addition of 15 μM DiOC_2_ (3) (Thermo Fisher) and incubation at room temperature for 5 min. Cells were then analysed on a BD LSR-Fortessa (BD Biosciences) using FACSDiva Software version 8.0.1 with 100,000 post-gating events recorded for each sample. Red/green ratios were calculated using the Ratio Tab of the FACSDiva by dividing the signal of 488B (red) by the signal of 488C (green) and multiplying by a percentage of the toal resolution. Ratios are calculated from uncompensated linear data and are always reported as linear data.

### Heterologous expression and purification of Cap1-C-terminal-His_6_-tagged construct.

Full-length Cap1-C-terminal-His_6_-tagged construct was expressed in *E. coli* Rosetta^™^ 2 (DE3) cells (Novagen). The freshly transformed cells were grown overnight as a primary culture at 37°C in Lysogeny broth (LB) media. The primary culture was further inoculated in terrific broth (TB) media and grown at 37°C till 1.2–1.3 OD_600nm_ and induced with 1 mM isopropyl β-D-1-thiogalactopyranoside (IPTG). The culture was further grown overnight at 16°C. The freshly harvested cells were resuspended in the lysis buffer (25 mM Hepes pH 8, 500 mM NaCl, 2 mM β-Mercaptoethanol and 5 % glycerol) supplemented with cOmplete mini, EDTA-free protease inhibitor tablets (Sigma). The resuspended cells were lysed by sonication at 50 % amplitude and 1 s on and 2 s off pulse for a total of 15 minutes. The unlysed cell debris were separated by centrifugation. The supernatant was used for harvesting the bacterial cell membrane. The membrane proteins were extracted from the purified bacterial cell membrane using 20 mM DDM (n-Dodecyl β-D Maltopyranoside) (Anatrace) detergent. The undissolved membrane was further separated using centrifugation and the supernatant was loaded on a pre-equilibrated (using lysis buffer with 1 mM DDM) 5 ml HisTrap column (Cytiva). The column was washed extensively with the lysis buffer supplemented with 40 mM imidazole and 1 mM DDM. The protein was eluted using the lysis buffer supplemented with 300 mM imidazole and 1 mM DDM. The protein fractions were checked on NuPAGE^™^ 4–12 % Bis-Tris gel (Invitrogen) and the pure fractions were pulled together and concentrated to load on the Superdex200 10/300-increase column. The equilibration buffer and the running buffer used to perform size exclusion chromatography was 25 mM Hepes pH 8, 200 mM NaCl, 2 mM β-Mercaptoethanol, 5 % glycerol supplemented with 375 μM GDN (glycol-diosgenin, Anatrace) detergent.

### SEC-MALS analysis with Cap1-C-terminal-His_6_-tagged construct.

The purified Cap1-C-terminal-His_6_-tagged sample was used for conjugate SEC-MALS analysis to determine the oligomeric state of the protein. The SEC-MALS experiment was performed using AKTA-Pure UV detector connected to SEC-MALS instrument (Wyatt) which has multi-angle light scattering detector and refractive index detector. Superdex200 10/300-increase column was used to run the protein sample in 25 mM Hepes pH 8, 200 mM NaCl, 2 mM β-Mercaptoethanol, 5 % glycerol and 375 μM GDN buffer. ASTRA 6 software was used for data analysis using protein conjugate method. The UV signal of the AKTA-Pure instrument was converted to analogue signal with a conversion factor of 1,000 mAU = 1 V. For protein conjugate analysis the refractive index increment (dn/dc) value for the protein was 0.185 ml g−1 and for the detergent was 0.143 ml g−1.

### Isothermal Titration Calorimetry (ITC) binding studies

ITC binding experiments were performed with 20 μM of His_6_-TEVsite-CARFL protein and its mutants titrated with 150 μM of cA_4_. The protein and the ligand were present in 25 mM Hepes pH 8, 200 mM NaCl, 2 mM β-mercaptoethanol and 5 % glycerol. MicroCal PEAQ-ITC (Malvern) instrument was used to perform the ITC studies at 25° C temperature. The protein was titrated with a total of 19 injections of cA_4_ out of which the volume of the first injection was 0.4 μl with a 0.8 s duration and rest of the 18 injections were 2 μl with a 4 s duration. The spacing between each of the injections was 150 s and the stirring speed was 750 rpm. The data was analyzed, and fitting was performed with MicroCal PEAQ-ITC Analysis software (Malvern) using one set of sites binding model and keeping number of binding sites (N) value constant to 1. The estimated *K*_d_ value for the CARFL WT (wild type) was ~33 nM. The estimated ΔH (kcal/mol), ΔG (kcal/mol) and -TΔS (kcal/mol) values were −3.21, −10.2 and −6.99 respectively. The estimated *K*_d_ value for the CARFL (Q118A) mutant was 1.2 mM ± 0.6 mM. The estimated ΔH (kcal/mol), ΔG (kcal/mol) and -TΔS (kcal/mol) values were −5.07 ± 0.621, −8.07, −3 respectively. No binding curve could be fitted in the case of CARFL(Q119A) mutant.

### Cryo-EM sample preparation and imaging

The peak fraction of the purified apo-Cap1 protein was concentrated to 150 μM (calculated for monomeric Cap1) and supplemented with 800 μM of cA_4_ ligand (incubated for 30 minutes on ice). This sample was used for the cA_4_-Cap1 grid preparation. In the case of apo-Cap1, the protein was concentrated to 200 μM and used for grid preparation in the presence of 0.5 mM FOM (Fluorinated Octyl Maltoside) detergent. The UltratiFoil Au R (1.2/1.3) grids were used for both the samples. The grids were glow discharged for 2 minutes at 15 mA. Multiple grids were frozen at 4°C, 100 % humidity, 12 s wait time, 3.5 – 4.5 s blot time and 0 blot force using Vitrobot Mark IV (FEI). Both apo Cap1 and cA_4_-Cap1 datasets were collected using Krios G4 microscope at MSKCC equipped with a Falcon 4i detector and an energy filter of 10 eV slit width. The pixel size was 0.725 Å and the defocus range used for these datasets was −0.8 μm to −2.3 μm. The images were collected in the EER mode with a total electron dose of 59.33 electrons per Å^2^ with an exposure rate 11.5 electrons/pixel/sec. The EER unsampling factor was 1 and EER number of fractions were 45.

### Cryo-EM data processing and refinement

We used cryoSPARC v4.4.1^38^ for the processing of apo Cap1 and cA_4_-Cap1 datasets (Table S1).

#### Processing of apo-Cap1 tetramer dataset

We collected 11,929 movies and performed Patch Motion Correction and Patch CTF estimation jobs. 4,795,274 particles were picked using blob picker job with blob picking parameters 150 – 200 Å. The particles were extracted with a box size of 400 pixel. Iterative 2D classification jobs were performed, and 207,755 particles were selected (Figs. S5A–B). Three ab-initio models were generated using ab-initio job (Fig. S5C). Out of the three models, the model (98,358 particles) marked by red box was used for the next refinement steps. Non-uniform refinement job was used to refine the map to 3.4 Å with C1 symmetry (Fig. S5D). The final set of particles used for the cryo-EM map was well distributed to different orientations (Fig. S5E) and the global resolution of the map was 3.4 Å following the standard FSC cutoff value of 0.143 (Fig. S5F). Further, local resolution values were estimated for the cryo-EM map of apo-Cap1 (Fig. S5G). The AF3^39^ models for each domain was used for the preliminary rigid body fitting using Chimera^40^ and the model was manually built using Coot^41^. Phenix^42^ real-space refinement program was used to remove the outliers and refine the model with a model vs. data correlation value (CC mask) of 0.84 (Table S1). In this cryo-EM map we observed densities to model two alternate conformations of the side chain of tyrosine 75 (Y75) residue of the TM1-TM2 segment (Figs. S6A–B). The model with flipped in Y75 side chain was refined with a model vs. data correlation value (CC mask) of 0.85 (Table S1). The model building for the cytosolic domain is displayed in Figures S6C–D.

#### Processing of cA_4_-Cap1 tetramer complex dataset

10,668 movies were collected for cA_4_-Cap1 sample and Patch Motion Correction and Patch CTF estimation jobs were performed using cryoSPARC. Blob picker job was used to pick up 42,443 particles from 1,061 micrographs. We selected 6,659 particles by 2D classification job to train a picking model using 1,061 micrographs to reduce the computation time using Topaz train job. The Topaz extract job was able to pick up 2,158,916 particles from 10,668 micrographs guided by previously trained model. 400 pixel box size was used for the particle extraction. Iterative 2D classification job was performed to select 325,786 particles (Figs. S7A–B). The particles were classified into three classes by 3D classification job and the class with 98,640 particles which showed all eight TM helices clearly was selected for the refinement job (Fig. S7C, red box). Non-uniform refinement was performed to resolve the map to 3.4 Å resolution with C1 symmetry (Fig. S7D). Another round of non-uniform refinement was performed with imposed C4 symmetry to improve the map quality and resolution specially at the TM domain (Fig. S7E). We observed good particle distribution both in the case of C1 and C4 symmetry maps (Figs. S7F–G). The standard FSC cut-off at 0.143 displayed 3.4 Å and 2.9 Å global resolution for C1 and C4 symmetry maps respectively (Figs. S7H–I). The cryo-EM map with C4 symmetry displayed that TM1-TM2 segment (Fig. S6E) and CARFL domain (Fig. S6F) was resolved with high resolution. Local resolution estimation on C1 symmetry map displayed that CARFL domain was resolved with high resolution even though the TM domain was poorly resolved (Fig. S7J). The local resolution estimation on the C4 symmetry-imposed map showed significantly improved resolution of the TM domain (Fig. S7K). AF3^39^ models of different domains were used for the rigid body fitting of the model using Chimera^40^. The model was further built using Coot^41^ and refined using real space refinement program in Phenix^42^ with a model vs. data correlation value (CC mask) of 0.85 (Table S1).

We re-processed 10,668 micrographs and picked up 4,629,563 particles using blob picker job in cryoSPARC. We extracted 3,838,293 particles using 400 pixel box size and performed iterative 2D classification jobs and screened 443,677 particles (Fig. S8A). Next, we built three ab-initio models out of which one model with 148,422 particles displayed the DUF domain (Figs. S8B–C). We used 3D classification job to classify 148,422 particles into five classes (Fig. S8D), out of which in one class the DUF domain was visible (Fig. S8D, red box). Next, we performed non-uniform refinement job with C1 symmetry which refined the map at 6.7 Å resolution (Fig. S8E). We imposed C4 symmetry in the next round of non-uniform refinement which improved the global resolution to 3.6 Å resolution (Fig. S8F). We observed good angular distribution for the particles used for final map generation (Fig. S8G). The FSC-plot showed 3.6 Å resolution at standard FSC (tight mask) cut-off 0.143 (Fig. S8H). The local resolution estimation of the map displayed even though the CARFL (Fig. S6G) and TM2 domain (Fig. S6H) was well resolved, the DUF has poor resolution (Fig. S6I). The TM1 and the loops were not resolved (Fig. S8I). We used AF3^39^ models for the rigid body fitting of the model using Chimera^40^. The model was further built using Coot^41^ and refined using real space refinement program in Phenix^42^ with a model vs. data correlation value (CC mask) of 0.73 (Table S1).

## Supplementary Material

Supplement 1

1
