# Supplementary material for: Cap1 forms a cyclic tetra-adenylate-induced membrane pore during the type III-A CRISPR-Cas immune response
Source: bioRxiv. 2025 Nov 13:2025.11.13.688252. Preprint. [Version 1] doi: 10.1101/2025.11.13.688252 (PMC12642688; doi:10.1101/2025.11.13.688252)
Supplement: 1 [file NIHPP2025.11.13.688252V1-supplement-1.pdf]

bioRxiv preprint doi: https://doi.org/10.1101/2025.11.13.688252; this version posted November 13, 2025. The copyright holder for this preprint (which was not certified by peer review) is the author/funder, who has granted bioRxiv a license to display the preprint in perpetuity. It is made available under aCC-BY 4.0 International license.

SUPPLEMENTARY FIGURE LEGENDS

Figure S1. Cap1 activation and toxicity in staphylococci. (A) Comparison of the III-A systems of *P. terrae* and *S. epidermidis* RP62a. Black boxes indicate CRISPR repeats; colored, numbered boxes indicate CRISPR spacers. Numbers indicate the percent homology or identity (in parentheses) of conserved Cas proteins. **(B)** Genetic modifications of the *S. epidermidis* RP62a type III-A CRISPR locus cloned into various pCRISPR plasmids. Amino acid substitutions, domain deletions, and insertion of different spacer sequences are indicated. **(C)** Growth of staphylococci carrying pTarget and diverse pCRISPR variants, measured as OD_{600} after the addition of aTc. Dotted line crosses at 360 minutes, the time used to generate bar graphs that represent the the different growth rates. **(D)** Chromatogram obtained after size exclusion purification of wild-type Cap1-CARFL-His_6. **(E)** SDS-PAGE followed by Coomassie-blue staining of purified wild-type and mutant CARFL Cap1-CARFL-His_6 proteins.

Figure S2. Apo-Cap1 structure. (A) SDS-PAGE followed by Coomassie-blue staining of peak fractions of purified full-length apo-Cap1 (34 kDa). **(B)** Representative 2D class averages showing different views of the apo-Cap1 protein. Class *a* displays a front view with cytosolic domains on top. Class *b* displays a side view in which the DUF4579, CARFL and TM1/2 domains are labelled. **(C)** Ribbon diagram of the apo-Cap1 tetramer showing in light grey the density for the GDN detergent micelle surrounding the transmembrane domains; the detergent density is marked by dotted lines. **(D)** Cryo-EM map of the apo-Cap1 tetramer. TM1 and TM2 are displayed in green and blue and form outer and inner rings, respectively, that insert into the bacterial membrane (beige background, which replaces the density observed for the GDN detergent micelle). The N-terminal segment (N, grey), CARFL domain (pink), the interconnecting loop (L, grey) and the DUF4579 domain (red) locate on the cytosolic side. **(E)** Cryo-EM map of apo-Cap1 viewed from the extracellular side showing extra density inside the pore, protruding inwards from one of the inner TM2 helices. The inner TM2 ring is delimited by the black box. **(F)** Density for the TM1 and TM2 helices shown in blue and green mesh; the black arrow indicates the two alternate conformations of the Y75 side chain. **(G)** Tetrameric arrangement of the inner TM2 ring viewed from the cytosolic side displaying the two alternate conformations of Y75 for one out of the four TM2 helices. The flipped Y75 side chain is shown in green. **(H)** Electrostatic surface representation of the cytosolic side view of the TM1 and TM2 (with Y75 side chain flipped in) domains, showing a closed pore. **(I)** Cytosolic view of the tetrameric CARFL domain of apo-Cap1, arranged in 4-fold symmetry. Alpha helix 1 (αH1) of one of the CARFL domain is labelled. **(J)** Electrostatic surface representation of the cytosolic side view of the tetrameric CARFL of apo-Cap1, showing a positively charged pocket at the center. **(K)** Conventional CARF monomer domain from apo-Cad1 protein (PDBID 9C77) showing a β-sheet formed by five parallel β-strands (1-5) and one antiparallel β-strand (6) sandwiched between two pairs of alpha helices, αH1-αH2 and αH3-αH4. **(L)** CARFL monomeric domain of apo-Cap1 showing a β-sheet composed of two parallel β-strands (1 and 3) and three antiparallel β-strands (2, 4, 5) sandwiched between a pair of alpha helices, αH1 and αH2. **(M)** Growth of staphylococci carrying pTarget and diverse pCRISPR variants harboring alanine substitutions of Cap1 residues shown in Figure 2F, measured as OD_{600} after the addition of aTc. Dotted line crosses at 360 minutes, the time used to generate bar graphs that represent the the different growth rates. **(N)**

bioRxiv preprint doi: https://doi.org/10.1101/2025.11.13.688252; this version posted November 13, 2025. The copyright holder for this preprint (which was not certified by peer review) is the author/funder, who has granted bioRxiv a license to display the preprint in perpetuity. It is made available under aCC-BY 4.0 International license.

Interacting residues of the CARFL tetramer showing interactions between the side chains of residues Q123, R165,F132, D112, R114 and Q115. **(O)** Growth of staphylococci carrying pTarget and diverse pCRISPR variants harboring alanine substitutions of Cap1 residues shown in Figure 2I, measured as OD_{600} after the addition of aTc. Dotted line crosses at 360 minutes, the time used to generate bar graphs that represent the different growth rates.

Figure S3. cA_4-bound Cap1 structure. (A) Cryo-EM map of the cA_4-Cap1 tetramer. TM1 and TM2 are displayed in green and blue and form outer and inner rings, respectively, that insert into the bacterial membrane (beige background, which replaces the density observed for the GDN detergent micelle; “Ex.”, extracellular space, “Cy.” Cytosol). The N-terminal segment (N, grey) and the CARFL domain (pink) locate on the cytosolic side. Density for the DUF4579 domain was not detected. **(B)** Size exclusion chromatograms of purified cA_4-bound Cap1 (red) and apo-Cap1 (black). The peak corresponding to unbound excess cA_4 is labelled. **(C)** Structure of the tetrameric CARFL domain of cA_4-bound Cap1 (without visible DUF4579) displaying the αH1 helices forming the cA_4 binding pocket. The density of the cA_4 molecule is shown in yellow from C1 symmetry map. **(D)** Same as (C) with the cA_4 density displayed from a C4 symmetry-imposed map. **(E)** Cad1-CARF dimer with bound cA_4 (PDB ID: 9C77). The base of the cA_4 binding pocket is formed by a pair of αH4 helices of each monomer, indicated by black arrows. **(F)** Side view of bound cA_4 within the CARFL pocket, showing adenine bases pointing upwards, not in the same plane as the rest of the molecule. **(G)** Tetrameric arrangement of C-terminal cA_4 binding domain of Csx23 (PDB 8QJK) with bound ligand. **(H)** The domain architecture of Csx23 protomer. **(I)** Chromatogram obtained after size exclusion purification of Cap1-CARFL^{Q118A}-His$_6$. **(J)** Same as (G) for Cap1-CARFL^{Q118A}-His$_6$. **(K)** Cryo-EM map of the cA_4-Cap1 tetramer. TM1 is not visible. TM2 is displayed in blue and forms an inner ring that inserts into the bacterial membrane (beige background, which replaces the density observed for the GDN detergent micelle; “Ex.”, extracellular space, “Cy.” Cytosol). The N-terminal segment (N, grey), CARFL domain (pink), the linker (L, grey) and the DUF4579 domain (red) locate on the cytosolic side. **(L)** CarfL domain residues interacting with cA_4 in the cA_4-bound Cap1 structure with a visible DUF4579. **(M)** Cytosolic view of the cA_4-bound Cap1 structure with a visible DUF4579. cA_4 (yellow) can be seen bound to the center of 4-fold symmetry of the CARFL domain, behind the DUF4579 helix-turn-helices tetramer.

Figure S4. Conformational changes of Cap1 upon cA_4 binding lead to pore opening and membrane depolarization. (A) Pore within the transmembrane region of apo-Cap1 structure (Y75 side chain flipped-in), predicted using Mole2.5 software. The bacterial membrane is represented by the beige background; “Ex.”, extracellular space, “Cy.” Cytosol. The inner volume of the pore is displayed by the orange mesh. The narrowest part of the pore is surrounded by four T64 residues belonging to inner TM2 helices. The predicted pore fails to cross the full membrane layer. **(B)** Growth of staphylococci carrying pTarget and diverse pCRISPR variants harboring alanine substitutions of Cap1 residues shown in Figure 4G, measured as OD_{600} after the addition of aTc. Dotted line crosses at 360 minutes, the time used to generate bar graphs that represent the the different growth rates. **(C)** Same as (B) for the E46R substitution. **(D)** Flow cytometry of *S. aureus* cells harboring various pCRISPR constructs and stained with DiOC2(3), collected 30 minutes after addition of aTc or the

bioRxiv preprint doi: https://doi.org/10.1101/2025.11.13.688252; this version posted November 13, 2025. The copyright holder for this preprint (which was not certified by peer review) is the author/funder, who has granted bioRxiv a license to display the preprint in perpetuity. It is made available under aCC-BY 4.0 International license.

depolarizing agent CCCP. “a.u.”, arbitrary units; green fluorescence, emission of 515 nm upon 488C excitation; red fluorescence, emission of 586 nm upon 488B excitation. Each plot is representative of approximately 100,000 cells. **(E)** Inward movement of αH1 helices of the CARFL domain upon cA_4 binding, indicated by magenta arrows. **(F)** Superposition of the top four AF3 models for apo-Cap1, showing a good agreement across all domains, including the DUF4579 domain (dotted rectangle). The bacterial membrane is represented by the beige background; “Ex.”, extracellular space, “Cy.” Cytosol. **(G)** Superposition of the helix-turn-helices of the tetrameric DUF4579 experimentally determined by cryo-EM (red) with the structure predicted by the top AF3 model (grey), showing a good alignment with RMSD 0.5 Å. **(H)** Superposition of the top four AF3 models for cA_4-Cap1, showing a good agreement across all domains, except the DUF4579 domain that displayed heterogenous structures (dotted rectangle). The bacterial membrane is represented by the beige background; “Ex.”, extracellular space, “Cy.” Cytosol. **(I)** Superposition of cA_4-Cap1 structure with visible DUF4579 and apo-Cap1, showing a 1.3 Å RMSD value. The bacterial membrane is represented by the beige background; “Ex.”, extracellular space, “Cy.” Cytosol. **(J)** Superposition of cA_4-Cap1 with and without visible DUF4579, showing a 2.4 Å RMSD value. The bacterial membrane is represented by the beige background; “Ex.”, extracellular space, “Cy.” Cytosol. Blue arrows, inward movement of TM1/2 domain; pink arrows, outward movement of the CARFL domain in DUF4579–cA_4-Cap1 structure relative to cA_4-Cap1 lacking structured DUF4579 in the cA_4-Cap1 structure with a visible DUF4579, relative to cA_4-Cap1 lacking structured DUF4579. **(K)** Comparison of TM2 domains of apo (grey), cA_4-Cap1 with visible DUF4579 (light blue) and cA_4-Cap1 without visible DUF4579 (dark blue). The bacterial membrane is represented by the beige background; “Ex.”, extracellular space, “Cy.” Cytosol. Blue arrows show TM2 helices moving inward in DUF4579–cA_4-Cap1 relative to cA_4-Cap1 lacking structured DUF4579. **(L)** Same as (A) for the cA_4-Cap1 with visible DUF4579 structure.

Figure S5. Cryo-EM data processing for apo-Cap1 protein. (A) Data processing steps including particle picking and 2D classification for the apo-Cap1 protein sample. The particle numbers are mentioned for each step. **(B)** Representative 2D class averages with different views of the apo-Cap1 protein. **(C)** Three ab-initio models were generated with final selected particles by 2D classification. The first model was selected for next round of refinement process (red box). **(D)** Cryo-EM map refined by non-uniform refinement job with C1 symmetry. **(E)** Angular distribution of the particles used for the final map. **(F)** Fourier shell correlation (FSC) plot, with and without mask. FSC value of 0.143 was used as threshold for resolution determination. **(G)** Local resolution estimated map displayed in different views including side view, extracellular view and cytosolic view. The scale bar is represented in Å unit.

Figure S6. Density tracing in the cryo-EM map of apo- and cA_4-bound Cap1 protein. (A) Map of the apo-Cap1 TM1 and TM2 segments indicating the extra density for the alternate conformation of Y75 side chain (black arrow) at contour level ~5 RMS. T64 residue is labelled. **(B)** Same density map as (A) displaying Y75 side chain at flipped-in conformation. The flipped side chain of Y75 is colored in green. **(C)** Electron density map of part of the apo-Cap1 CARFL domain displayed at contour level ~5 RMS. **(D)** Same as (C) displaying for the apo Cap1 DUF4579 domain. **(E)** Electron density map of cA_4-Cap1 (without visible DUF4579) C4 symmetry-imposed map at contour level

bioRxiv preprint doi: https://doi.org/10.1101/2025.11.13.688252; this version posted November 13, 2025. The copyright holder for this preprint (which was not certified by peer review) is the author/funder, who has granted bioRxiv a license to display the preprint in perpetuity. It is made available under aCC-BY 4.0 International license.

~5 RMSD displaying representative TM1 and TM2 segments. Y75 and T64 residues are labelled. **(F)** Similar representation as in panel (E) showing map for part of the CARFL domain at contour level ~5 RMS. **(G)** Map of one of the TM2 domain from cA_4-Cap1-DUF4579 structure displayed at contour level ~5 RMS. **(H-I)** Similar representation of the maps as panel (G) for the CARFL and DUF at similar contour level.

Figure S7. Cryo-EM data processing for cA_4-Cap1 (without visible DUF) complex. **(A)** Steps used for cA_4-Cap1 data processing including particle picking by blob picker and Topaz, and 2D classification jobs. **(B)** Representative 2D class averages are displayed with different views of cA_4-Cap1 complex. **(C)** Cryo-EM maps generated using 3D classification job. The class marked with red box was used for next round of refinement. **(D)** Cryo-EM map of the cA_4-Cap1 complex refined by non-uniform refinement job with C1 symmetry. **(E)** The resolution of the map shown in (D) was further improved by another round of non-uniform refinement with C4 symmetry. **(F)** Angular distribution of the particles used in the non-uniform refinement with C1 symmetry. **(G)** Similar plot as in (F) in the case of the non-uniform refinement with C4 symmetry in displayed. **(H)** FSC plot with and without mask generated by non-uniform refinement with C1 symmetry. **(I)** Similar FSC plot as in (H) generated by non-uniform refinement with C4 symmetry. **(J)** Local resolution estimation of the C1 symmetry cryo-EM map of cA_4-Cap1 complex. **(K)** Similar as in (J) for C4 symmetry cryo-EM map of the cA_4-Cap1 complex. The scale bar is presented in Å unit for both maps in (J) and (K).

Figure S8. Cryo-EM data processing for cA_4-Cap1 (with visible DUF4579) complex. **(A)** Particles were picked up using blob picker and screened using iterative 2D classification job. **(B)** Representative 2D classes (6,102 particles) are displayed with visible DUF4579 domain. **(C)** The particles selected by 2D classification job were used to build ab-initio models. A model marked by red box displayed the DUF4579 domain. **(D)** This model corresponding to 148,422 particles was used for 3D classification job. Out of the five 3D classes, one class containing 28,046 particles displayed intact DUF4579 domain marked in red box. **(E)** Non-uniform refinement job was performed with C1 symmetry, that resolved the map at 6.7 Å resolution. **(F)** C4 symmetry imposed non-uniform refinement improved the global resolution to 3.6 Å. **(G)** Angular distribution of the particles used for the refinement jobs. **(H)** FSC plot generated by the non-uniform refinement job. **(I)** The local resolution estimation indicates that the CARFL and TM2 domains have resolution close to 3.5 Å but the DUF4579 domain was poorly resolved. Additionally, TM1 and the loops were not resolved. The scale bar is presented in Å unit.

bioRxiv preprint doi: https://doi.org/10.1101/2025.11.13.688252; this version posted November 13, 2025. The copyright holder for this preprint (which was not certified by peer review) is the author/funder, who has granted bioRxiv a license to display the preprint in perpetuity. It is made available under aCC-BY 4.0 International license.

917 **Table S1. Cryo-EM data collection, refinement, and validation statistics**

	Apo-Cap1 (Y75 flipped out) (PDB XXXX) (EMDB xxxxx)	Apo-Cap1 (Y75 flipped in) (PDB XXXX) (EMDB xxxxx)	cA_4-Cap1 (PDB XXXX) (EMDB xxxxx)	cA_4-Cap1 with DUF (PDB XXXX) (EMDB xxxxx)
**Data collection and Processing**				
Microscope	Krios G4	Krios G4	Krios G4	Krios G4
Voltage (keV)	300	300	300	300
Camera	Falcon 4i	Falcon 4i	Falcon 4i	Falcon 4i
Magnification	165000	165000	165000	165000
Pixel size at detector (Å/pixel)	0.725	0.725	0.725	0.725
Total electron exposure (e^-/Å^2)	59.33	59.33	59.33	59.33
Exposure rate (e-/pixel/sec)	11.5	11.5	11.5	11.5
EER number of fractions	45	45	45	45
EER unsampling factor	1	1	1	1
Defocus range (µm)	-0.8 to -2.3	-0.8 to -2.3	-0.8 to -2.3	-0.8 to -2.3
Phase plate (if used)	-	-	-	-
- phase shift range (in degrees)	-	-	-	-
- number of images per phase plate	-	-	-	-
Automation software	EPU	EPU	EPU	EPU
Tilt angle (if grid was tilted)	-	-	-	-
Energy filter slit width (eV)	10	10	10	10
Micrographs collected (no.)	11,929	11,929	10,668	10,668
Micrographs used (no.)	11,929	11,929	10,668	10,668
Total extracted particles (no.)	4,795,274	4,795,274	2,158,916	4,629,563
For each reconstruction:				
Refined particles (no.)	98,358	98,358	98,640	28,046
Final particles (no.)	98,358	98,358	98,640	28,046
Point-group or helical symmetry	-	-	-	-
Estimated error of translations/rotations	-	-	-	-
Resolution (global, Å)	3.4	3.4	2.9	3.6
FSC 0.5 (unmasked/masked)	-	-	-	-
FSC 0.143 (unmasked/masked)	4/3.4	4/3.4	3.4/2.9	4.3/3.6
Resolution range (local, Å)	3-7	3-7	3-7	3.5-7
Resolution range due to anisotropy (Å)	-	-	-	-
Map sharpening *B* factor (Å^2)	-	-	-	-
Map sharpening methods	-	-	-	-
**Model composition (for each model)**				
Protein	1109	1109	844	1005
Ligands	0	0	0	0
RNA/DNA	0	0	4	4
**Model Refinement (for each model)**				

bioRxiv preprint doi: https://doi.org/10.1101/2025.11.13.688252; this version posted November 13, 2025. The copyright holder for this preprint (which was not certified by peer review) is the author/funder, who has granted bioRxiv a license to display the preprint in perpetuity. It is made available under aCC-BY 4.0 International license.

Refinement package	Phenix	Phenix	Phenix	Phenix
- real or reciprocal space	Real space	Real space	Real space	Real space
- resolution cutoff	3.3	3.3	2.9	3.6
Model-Map scores				
-CC	0.84 (mask)	0.85 (mask)	0.85 (mask)	0.73 (mask)
- FSC (d FSC model at 0.143)	3.3 (mask)	3.3 (mask)	2.9 (mask)	3.5 (mask)
B factors (Å^2)				
Protein residues	142.45	142.67	141.11	99.96
Ligands	-	-	-	-
RNA/DNA	-	-	112.56	51.93
R.m.s. deviations from ideal values				
Bond lengths (Å)	0.003	0.002	0.005	0.003
Bond angles (°)	0.527	0.513	0.757	0.637
**Validation (for each model)**				
MolProbity score	1.56	1.57	2.48	1.88
CaBLAM outliers	0.74	0.93	2.96	0.65
Clashscore	10.92	11.46	13.57	15.78
Poor rotamers (%)	0.48	0.48	6.57	0.96
C-beta deviations	0	0	0	0
EMRinger score	-	-	-	-
Ramachandran plot				
Favored (%)	98.17	98.26	96.62	96.99
Outliers (%)	0.37	0.37	0.00	0.21

bioRxiv preprint doi: https://doi.org/10.1101/2025.11.13.688252; this version posted November 13, 2025. The copyright holder for this preprint (which was not certified by peer review) is the author/funder, who has granted bioRxiv a license to display the preprint in perpetuity. It is made available under aCC-BY 4.0 International license.

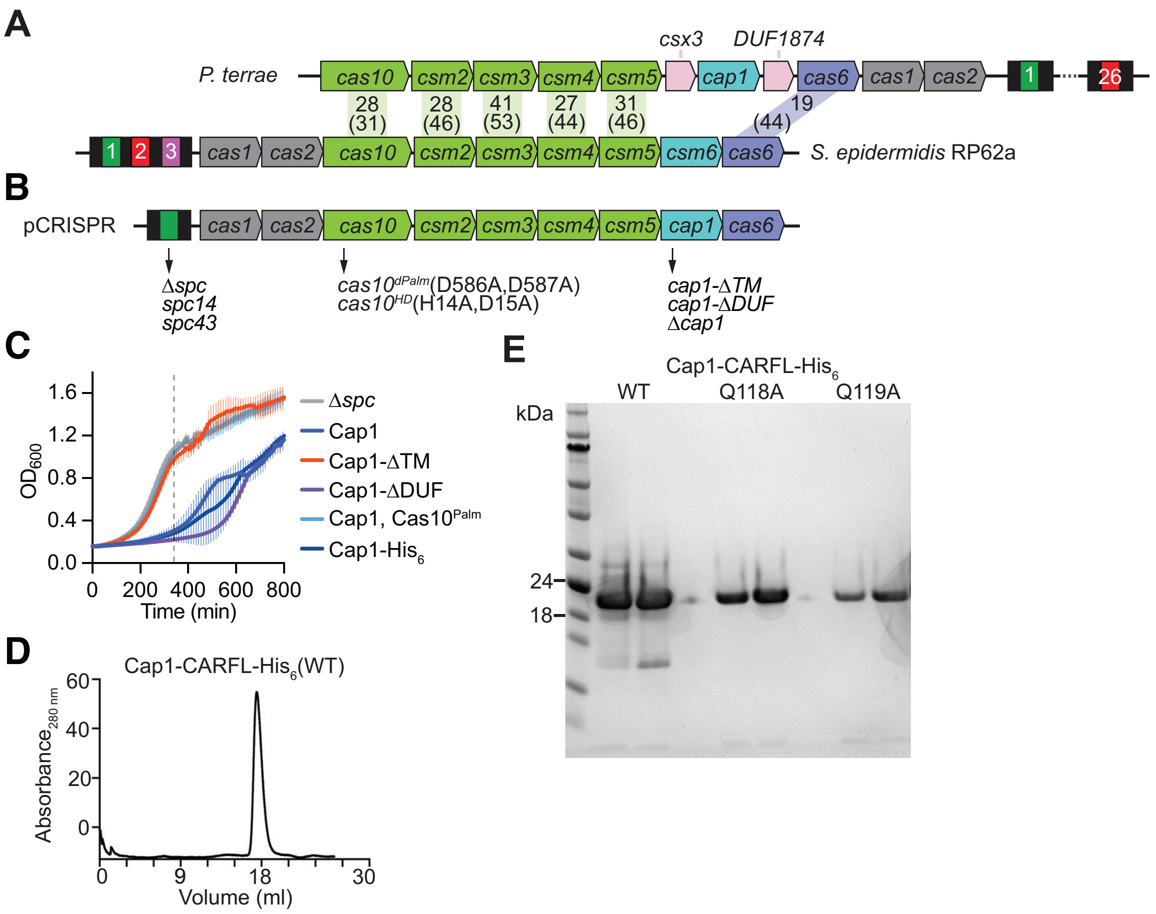

Fig. S1, Majumder, Cahir *et al*.

bioRxiv preprint doi: https://doi.org/10.1101/2025.11.13.688252; this version posted November 13, 2025. The copyright holder for this preprint (which was not certified by peer review) is the author/funder, who has granted bioRxiv a license to display the preprint in perpetuity. It is made available under aCC-BY 4.0 International license.

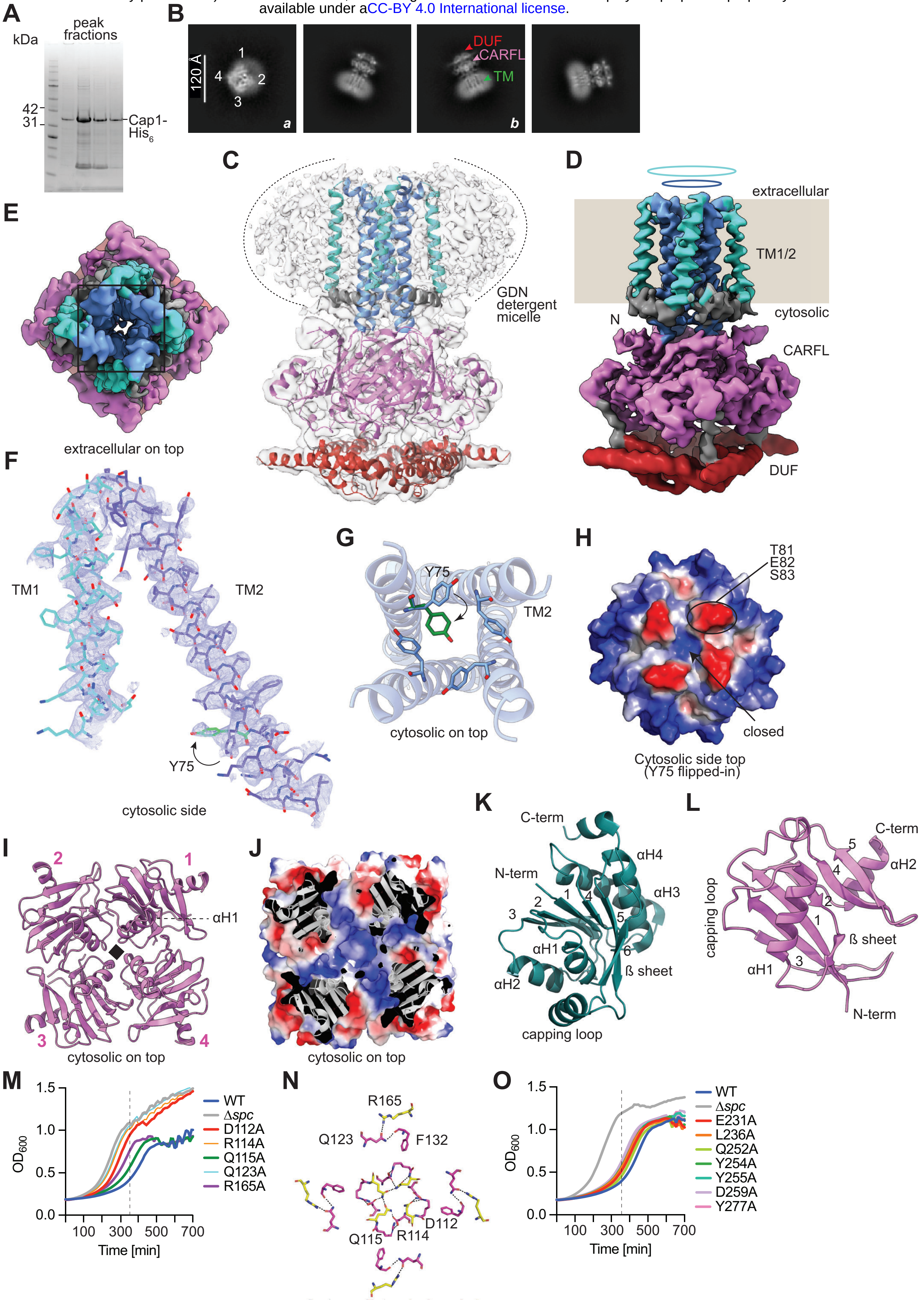

Fig. S2. Apo Cap1 structure

bioRxiv preprint doi: https://doi.org/10.1101/2025.11.13.688252; this version posted November 13, 2025. The copyright holder for this preprint (which was not certified by peer review) is the author/funder, who has granted bioRxiv a license to display the preprint in perpetuity. It is made available under aCC-BY 4.0 International license.

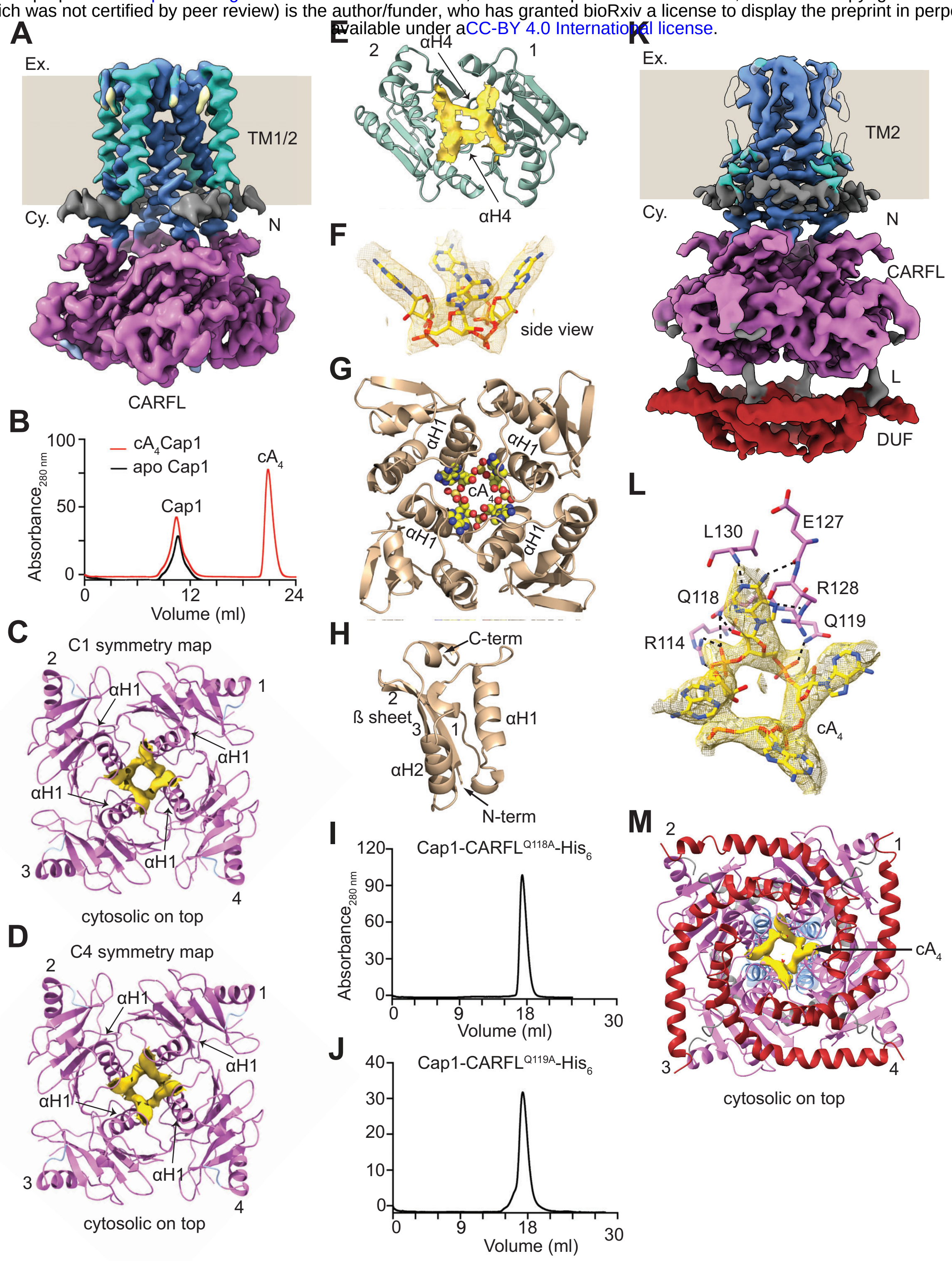

Fig. S3. cA4 bound Cap1

bioRxiv preprint doi: https://doi.org/10.1101/2025.11.13.688252; this version posted November 13, 2025. The copyright holder for this preprint (which was not certified by peer review) is the author/funder, who has granted bioRxiv a license to display the preprint in perpetuity. It is made available under aCC-BY 4.0 International license.

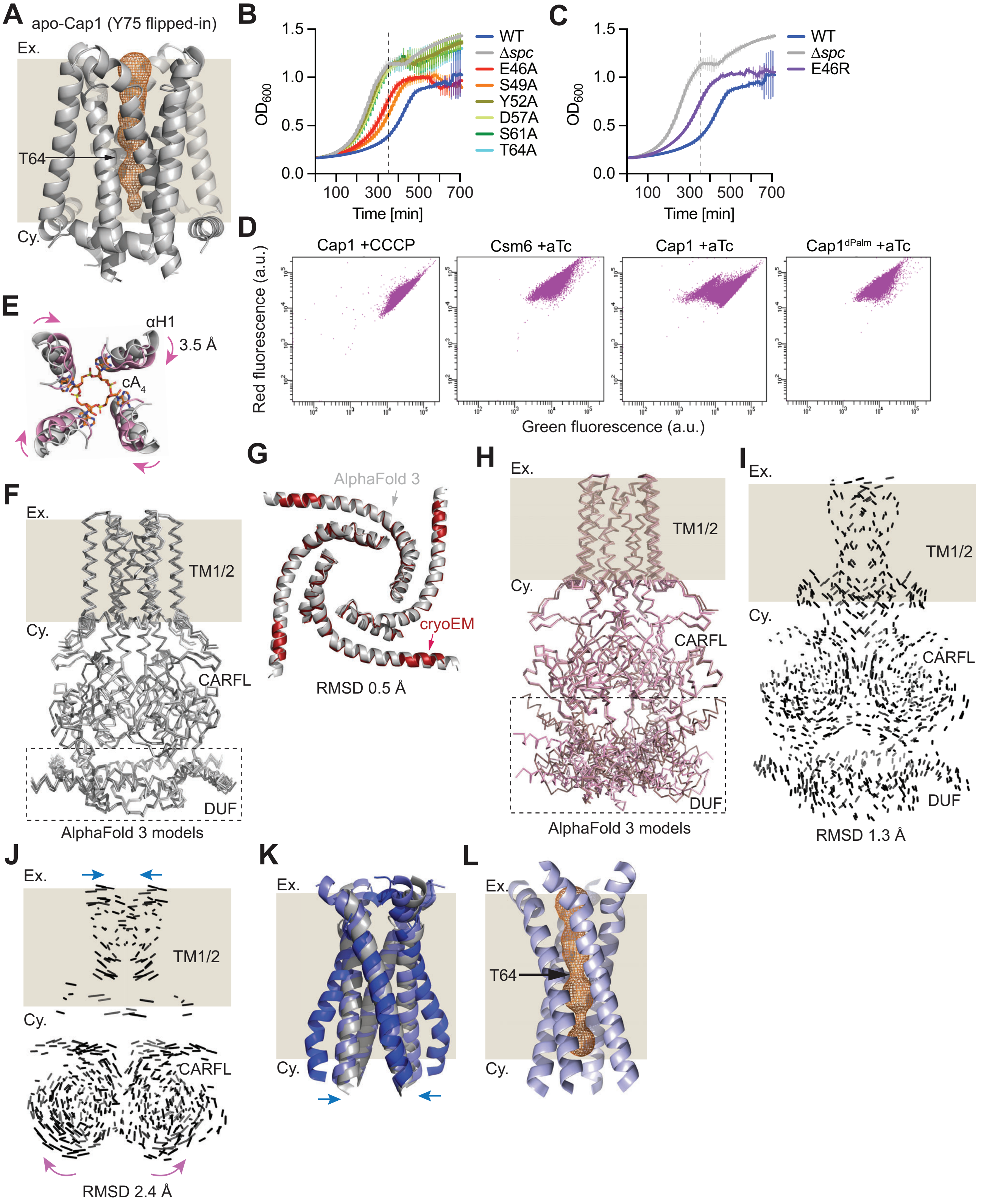

Fig. S4, Majumder, Cahir *et al*.

bioRxiv preprint doi: https://doi.org/10.1101/2025.11.13.688252; this version posted November 13, 2025. The copyright holder for this preprint (which was not certified by peer review) is the author/funder, who has granted bioRxiv a license to display the preprint in perpetuity. It is made available under aCC-BY 4.0 International license.

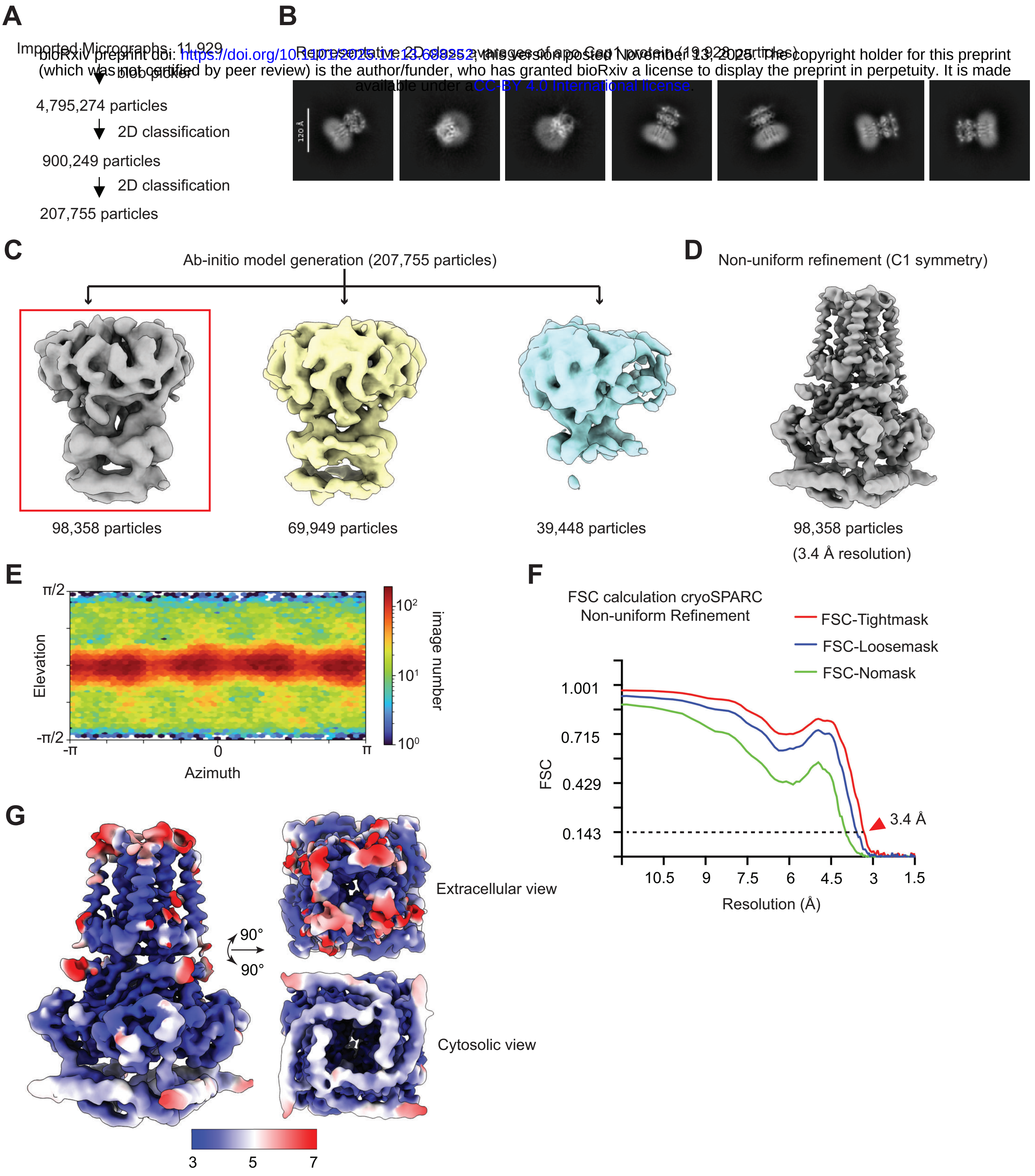

Fig. S5. Apo-Cap1 data processing

bioRxiv preprint doi: https://doi.org/10.1101/2025.11.13.688252; this version posted November 13, 2025. The copyright holder for this preprint (which was not certified by peer review) is the author/funder, who has granted bioRxiv a license to display the preprint in perpetuity. It is made available under aCC-BY 4.0 International license.

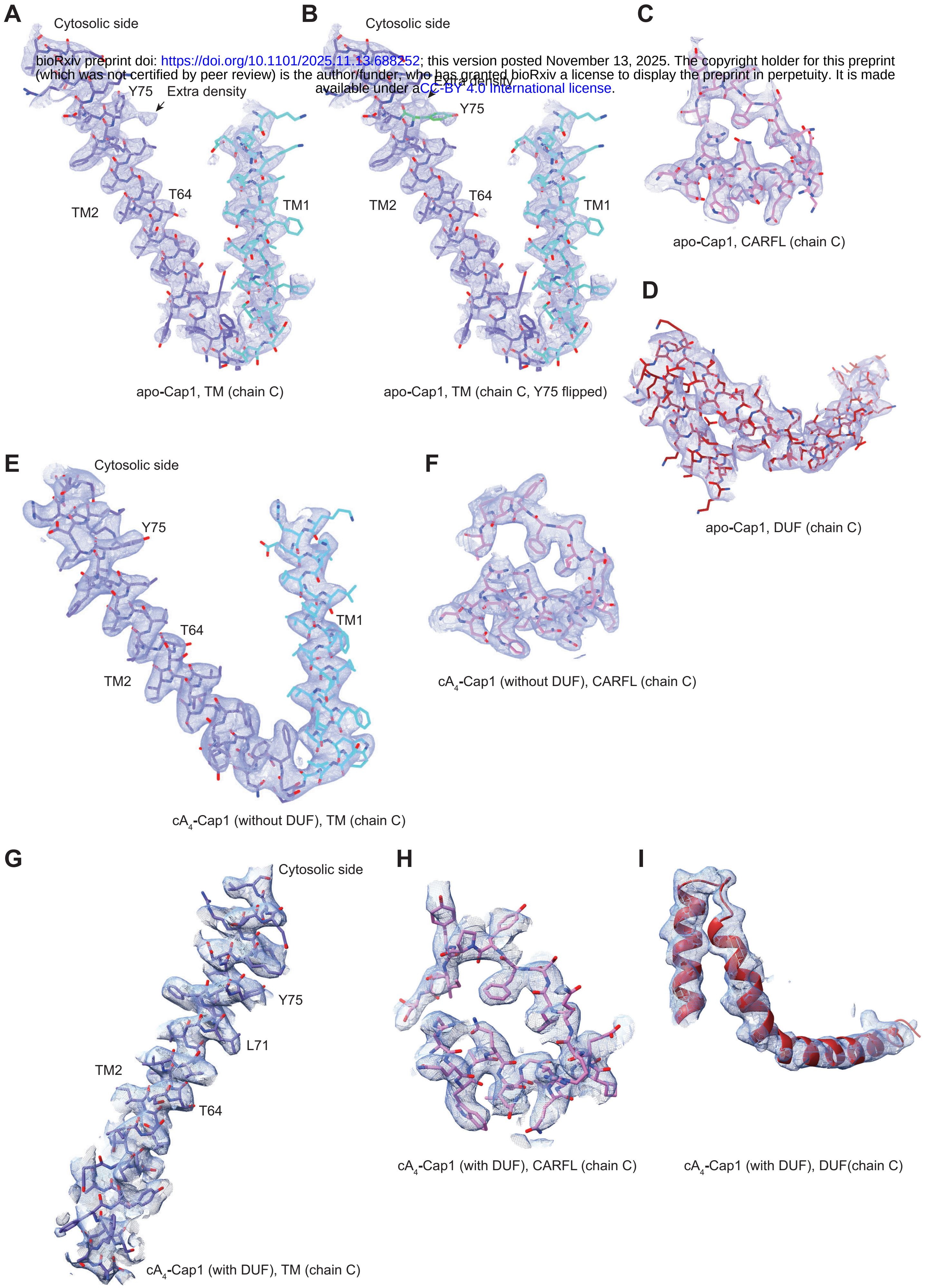

Fig. S6. cryo-EM map of Cap1 and cA4-Cap1

bioRxiv preprint doi: https://doi.org/10.1101/2025.11.13.688252; this version posted November 13, 2025. The copyright holder for this preprint (which was not certified by peer review) is the author/funder, who has granted bioRxiv a license to display the preprint in perpetuity. It is made available under aCC-BY 4.0 International license.

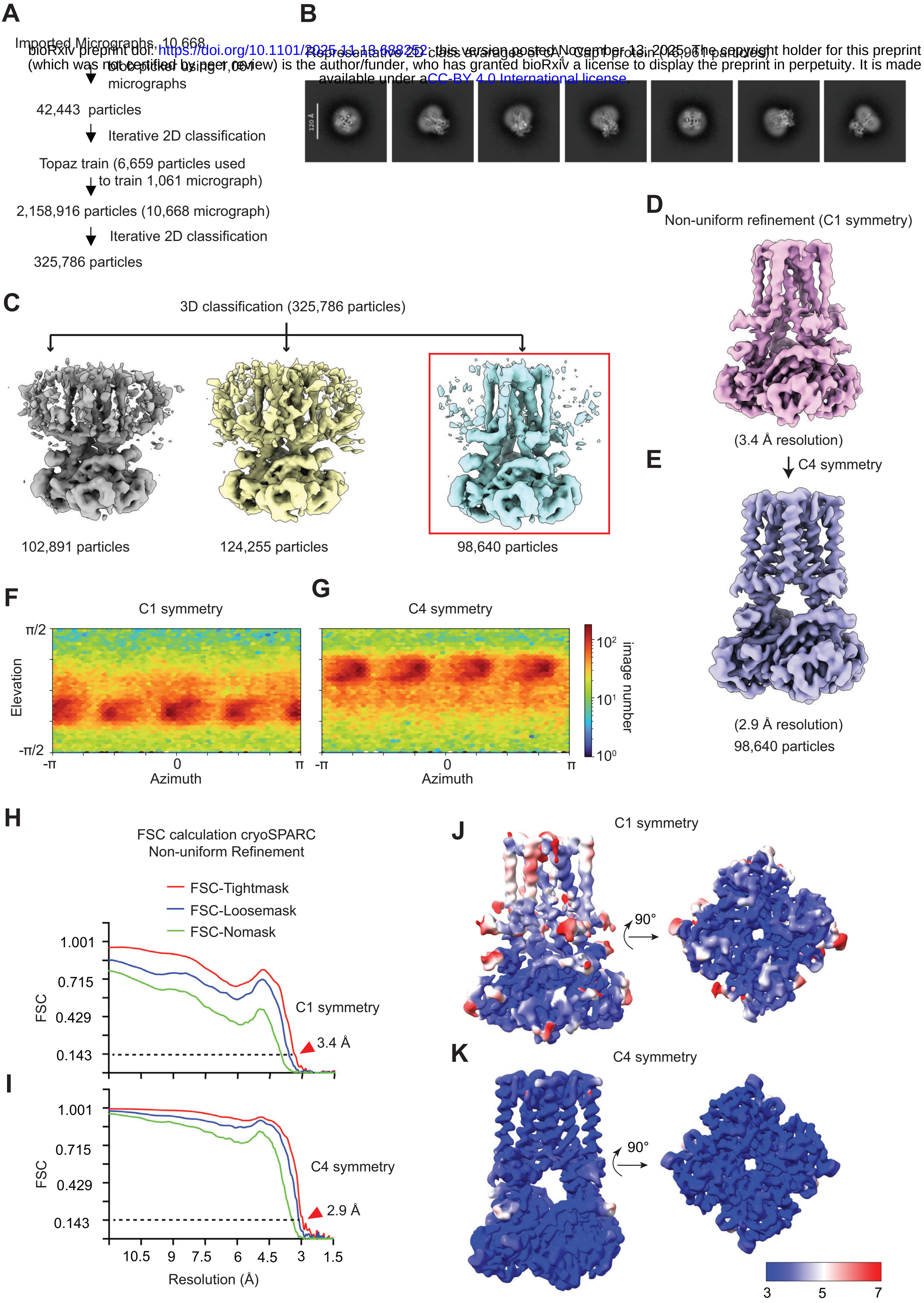

Fig. S7. cA4-Cap1 data processing

bioRxiv preprint doi: https://doi.org/10.1101/2025.11.13.688252; this version posted November 13, 2025. The copyright holder for this preprint (which was not certified by peer review) is the author/funder, who has granted bioRxiv a license to display the preprint in perpetuity. It is made available under aCC-BY 4.0 International license.

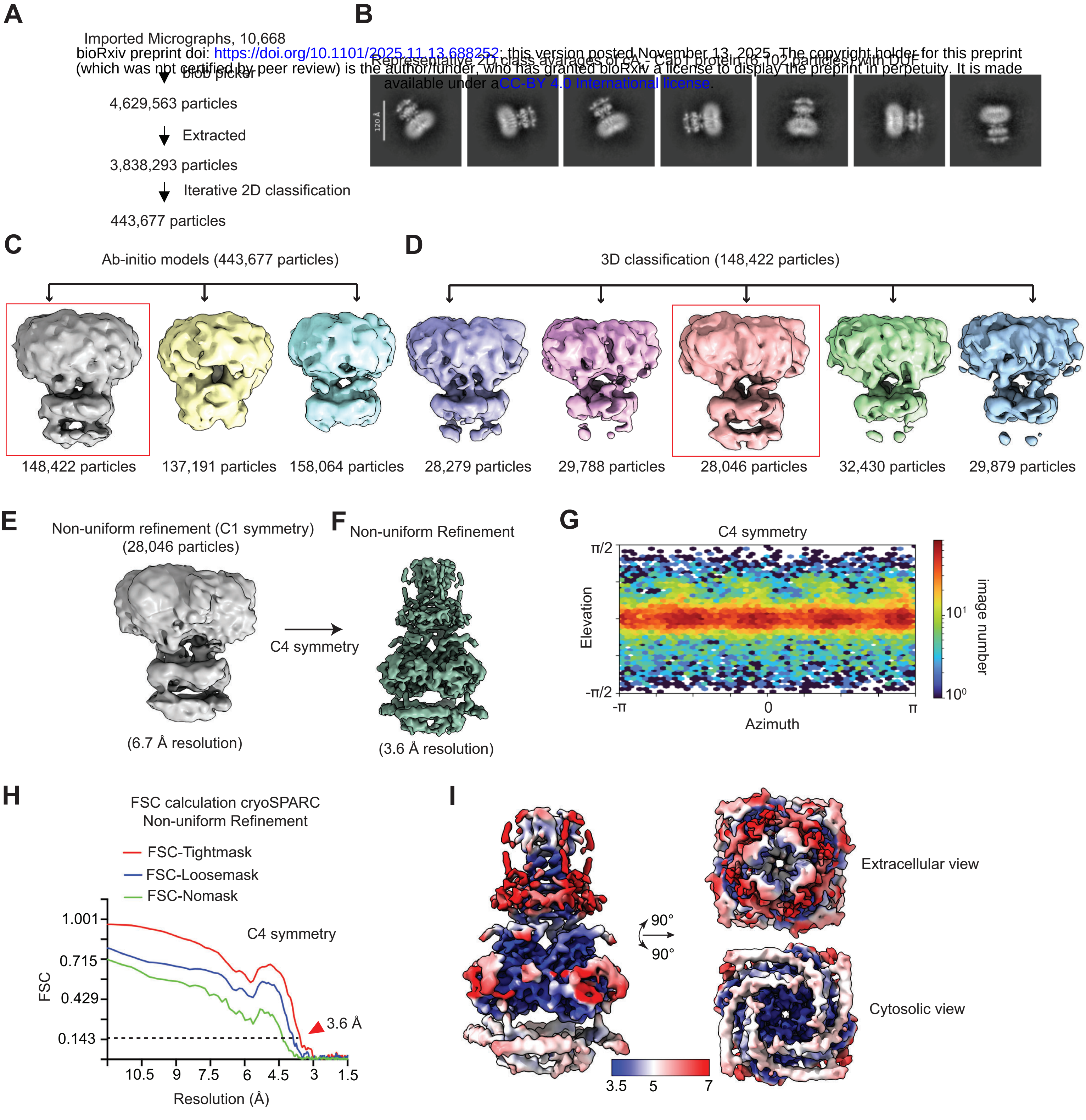

Fig. S8. cA4-Cap1 reprocessing reveals DUF-containing particle subset
